# Molecular epidemiology and population genetics of *Schistosoma mansoni* infecting school-aged children situated along the southern shoreline of Lake Malawi, Malawi

**DOI:** 10.1371/journal.pntd.0012504

**Published:** 2024-10-07

**Authors:** John Archer, Lucas J. Cunningham, Alexandra Juhász, Sam Jones, Angus M. O’Ferrall, Sarah Rollason, Bright Mainga, Priscilla Chammudzi, Donales R. Kapira, David Lally, Gladys Namacha, Peter Makaula, James E. LaCourse, Sekeleghe A. Kayuni, Bonnie L. Webster, Janelisa Musaya, J. Russell Stothard

**Affiliations:** 1 Department of Tropical Disease Biology, Liverpool School of Tropical Medicine, Liverpool, United Kingdom; 2 Wolfson Wellcome Biomedical Laboratories, Department of Zoology, Natural History Museum, Cromwell Road, London, United Kingdom; 3 Institute of Medical Microbiology, Semmelweis University, Budapest, Hungary; 4 School of Biosciences, University of Cardiff, Cardiff, United Kingdom; 5 Laboratory Department, Mangochi District Hospital, Mangochi, Malawi; 6 Malawi-Liverpool-Wellcome Trust Clinical Research Programme, Queen Elizabeth Central Hospital, Blantyre, Malawi; 7 Department of Pathology, School of Medicine and Oral Health, Kamuzu University of Health Sciences (KUHeS), Blantyre, Malawi; Food and Drug Administration, UNITED STATES OF AMERICA

## Abstract

**Background:**

In areas of low disease endemicity, highly sensitive diagnostic tools to identify, diagnose, and monitor intestinal schistosomiasis transmission are needed to reliably measure the burden and risk of infection. Here, we used highly sensitive molecular diagnostic methods to investigate *Schistosoma mansoni* prevalence and transmission along the southern shoreline of Lake Malawi, five years post-disease outbreak.

**Methodology and principal findings:**

Faecal and urine samples were provided by school-aged children situated along the southern shoreline of Lake Malawi. Kato-Katz faecal-egg microscopy and point-of-care circulating cathodic antigen (POC-CCA) rapid diagnostic tests were then performed to diagnose infection with *S*. *mansoni*. Urine-egg microscopy was also used to diagnose infection with *Schistosoma haematobium*. In addition, *Schistosoma* miracidia were isolated from faecal material using a standard miracidium hatching technique. A two-step real-time PCR approach was then used to diagnose infection with *S*. *mansoni* using DNA isolated from faecal samples. Furthermore, isolated miracidia were genotyped to species level through PCR and Sanger sequencing. Phylogenetic analyses were then carried out to identify which previously defined *S*. *mansoni cox*1 lineage group *S*. *mansoni* miracidia were most closely related to.

The measured prevalence of *S*. *mansoni* infection varied considerably depending on which diagnostic assay was used. When compared to real-time PCR, faecal-egg microscopy had a sensitivity of 9% and a specificity of 100%. When POC-CCA ‘trace’ results were considered positive, POC-CCA had a sensitivity of 73% and a specificity of 81% when compared to real-time PCR. However, when considered negative, POC-CCA sensitivity was reduced to 56%, whereas specificity was increased to 90%. In addition, a high degree of *S*. *haematobium* DNA was detected in DNA isolated from faecal samples and motile *S*. *haematobium* miracidia were recovered from faecal samples. *Schistosoma mansoni* miracidia were closely related to two independent *cox*1 lineage groups, suggesting multiple recent introduction and colonisation events originating from surrounding east African countries.

**Conclusions and significance:**

Intestinal schistosomiasis is now highly prevalent along the southern shoreline of Lake Malawi just five years post-disease outbreak. In addition, a high prevalence of urogenital schistosomiasis persists. The revision of ongoing schistosomiasis control programmes in this area is therefore recommended. Our study also highlights the need for reliable diagnostic assays capable of distinguishing between *Schistosoma* species in multispecies co-endemic areas.

## Author summary

Highly sensitive diagnostic approaches to identify, diagnose, and monitor intestinal schistosomiasis transmission are needed to reliably measure the burden and risk of infection in endemic areas. Here, we used a range of diagnostic approaches, including highly sensitive molecular diagnostic methods, to investigate *Schistosoma mansoni* prevalence and transmission along the southern shoreline of Lake Malawi, Mangochi District, Malawi, five years post-disease outbreak. The measured prevalence of *S*. *mansoni* infection varied considerably depending on which diagnostic assay was used. Additionally, a high degree of *Schistosoma haematobium* DNA was detected in DNA isolated from faecal samples and motile *S*. *haematobium* miracidia were recovered from faecal samples. Through phylogenetic analyses, it was also suggested that multiple introduction and colonisation events of *S*. *mansoni* originating from surrounding east African countries into Mangochi District may have recently taken place. As such, the revision of ongoing schistosomiasis control programmes in this area is recommended. Our study also highlights the need for reliable diagnostic assays capable of distinguishing between *Schistosoma* species in multispecies co-endemic areas.

## 1. Introduction

Schistosomiasis is a neglected tropical disease (NTD) caused by infection with parasitic trematodes of the genus *Schistosoma* that can lead to debilitating morbidity and mortality [1]. Whilst it is estimated that over 230 million people are currently infected globally, approximately 95% of all cases occur within sub-Saharan Africa [2]. Of these, around one-third are deemed intestinal schistosomiasis, caused predominantly by infection with *Schistosoma mansoni* but also less commonly by infection with *Schistosoma intercalatum* and *Schistosoma guineensis* in some restricted areas of central Africa [3,4]. The remaining two-thirds of schistosomiasis cases in sub-Saharan Africa are deemed urogenital schistosomiasis, caused by infection with *Schistosoma haematobium*. Both intestinal and urogenital forms of schistosomiasis differ considerably in their pathogenesis, and so species-specific diagnostic tools are needed in co-endemic areas [1].

Pathologies associated with intestinal schistosomiasis are caused primarily by the copious number of eggs produced by female adult worms which inhabit the mesenteric veins of the intestine [1]. To perpetuate the parasite’s life cycle, these eggs penetrate blood vessel walls and migrate through surrounding tissues with the aim of reaching the lumen of the intestine for excretion and onward transmission. A large proportion of eggs, however, are not excreted and instead become sequestered throughout the intestinal system and liver. This can evoke a T helper type-2 (Th2) cell-driven granulomatous response that often leads to chronic inflammation, severe abdominal pain, diarrhoea, portal hypertension, liver fibrosis and permanent organ damage [5].

Eggs that are successfully passed within the faeces will hatch into the first *S*. *mansoni* larval stage, miracidia, upon freshwater contact. Hatched miracidia will then actively seek and penetrate the soft tissues of obligate freshwater snail intermediate hosts of the genus *Biomphalaria* (Gastropoda: Planorbidae), where they will undergo asexual replication over a period of approximately 4–6 weeks [1]. Following this period, the second *S*. *mansoni* larval stage, cercaria, are shed from *Biomphalaria* intermediate hosts back into the surrounding freshwater. Shed cercariae will then actively seek and penetrate the skin of a suitable mammalian definitive host making contact with *S*. *mansoni*-contaminated freshwater, e.g., humans. Once within the definitive host, cercariae will mature into juvenile schistosomula which will then migrate through the circulatory system to the hepatic portal vein of the liver. Here, schistosomula mature into adults and pair with a mate. Paired adult schistosomes then migrate from the liver, against the flow of blood, to the mesenteric veins of the intestine. This migration within the definitive host takes approximately 6–8 weeks [6].

Intestinal schistosomiasis is typically diagnosed using Kato-Katz faecal-egg microscopy [7,8]. Whilst this method is relatively inexpensive and can be carried out at the point-of-care, it is considered low-throughput and lacks sensitivity, particularly when attempting to diagnose individuals harbouring low-intensity infections [8]. As a result, reliably measuring the prevalence of intestinal schistosomiasis in areas of low disease transmission and endemicity, such as those that have undergone repeated annual or bi-annual mass drug administration (MDA) with the anthelmintic drug praziquantel [9] or in areas where a recent outbreak of infections has occurred [10], can be extremely difficult using this method alone. For these reasons, a variety of immunological rapid diagnostic lateral flow tests (RDTs) have also been developed to diagnose infection with *S*. *mansoni*, of which the most widely used is the urine-based point-of-care circulating cathodic antigen (POC-CCA), [11]. This assay, however, can also lack sensitivity, as well as specificity, when attempting to diagnose individuals harbouring low-intensity *S*. *mansoni* infections or when assessing individuals co-infected with both *S*. *mansoni* and *S*. *haematobium* [12,13].

Whilst both Kato-Katz faecal-egg microscopy and POC-CCA assays are valuable diagnostic tools in certain endemic settings, highly sensitive molecular assays, such as endpoint and real-time PCR, are also needed for effective and impactful disease diagnosis and transmission monitoring in areas of low disease endemicity [14]. As such, their continued development and use has been recommended by the World Health Organisation (WHO) [15]. Additional advantages of developing molecular assays such as these include their capacity to be used to infer phylogenetic relationships between *Schistosoma* trematodes, allowing for the examination of intra- and inter-species population diversity and structuring, and for the identification of *Schistosoma* spp. hybrids [16,17]

Lake Malawi is one of seven African Great lakes. With an approximate water surface area of 29,600 Km^2^, Lake Malawi occupies approximately one-fifth of Malawi whilst also bordering Tanzania and Mozambique along its eastern shoreline. The lake’s most southern shoreline, however, borders Mangochi District; one of Malawi’s 28 districts. Owing to insufficient water, sanitation, and hygiene (WASH) infrastructure, many members of Mangochi’s multiple communities rely on the lake as a source of drinking water, food (*via* fishing), a place to bathe, a place to tend livestock, and for recreation. As such, human water-contact with the lake’s shoreline is commonplace, resulting in a high prevalence of waterborne diseases in this area, including intestinal schistosomiasis, urogenital schistosomiasis and giardiasis [10,18,19].

Until recently, intestinal schistosomiasis was not considered endemic in Mangochi District as no species of *Biomphalaria* freshwater snails were known to inhabit the southernmost shoreline of Lake Malawi. However, during routine malacological surveillance in November of 2017, discreet populations of *Bi*. *pfeifferi* snails were unexpectedly encountered in submerged beds of *Vallisneria* freshwater vegetation [20]. Subsequently, in May of 2018, an outbreak of intestinal schistosomiasis was confirmed despite annual and ongoing MDA campaigns in this area implemented to reduce transmission of urogenital schistosomiasis [10], and in May of 2019, a low prevalence of intestinal schistosomiasis was recorded in school-aged children using Kato-Katz faecal-egg microscopy (mean prevalence 6.4% across four schools), whereas a much higher prevalence was recorded using POC-CCA (79.6% mean prevalence when POC-CCA ‘trace’ results were considered positive and 23.7% mean prevalence when ‘trace’ results were considered negative, across the same four schools).

Here, we aimed to measure the prevalence of intestinal schistosomiasis in school-aged children situated along this southern shoreline of Lake Malawi using highly sensitive and specific molecular diagnostic approaches and assess the diagnostic performance of Kato-Katz faecal-egg microscopy and POC-CCA in an area co-endemic for both intestinal and urogenital schistosomiasis. We also aimed to identify which previously defined *S*. *mansoni* cytochrome oxidase subunit 1 (*cox*1) lineage group(s) (I–V) Mangochi District *S*. *mansoni* isolates were most closely related to [16]. The purpose of this study was to therefore gain a more thorough understanding of intestinal schistosomiasis transmission in Mangochi District five years post-outbreak to better inform and strengthen future disease control efforts.

## 2. Methods

### 2.1. Ethics statement

This study was carried out as part of the *Hybridisations in UroGenital Schistosomiasis* (HUGS) project [21]. Ethical approval and research authorisations were approved in the UK by the Liverpool School of Tropical Medicine (LSTM) Research Ethics Committee (application 17–018) and in Malawi by the National Health Sciences Research Committee (1805). Informed, written assent was obtained from the guardians of all child participants enrolled in the study prior to any data or clinical sample collection. Study participants who tested positive for schistosomiasis (intestinal or urogenital) by either faecal/urine-egg microscopy or POC-CCA were provided with the recommended dose of praziquantel according to participant height under the supervision of the study clinician (SAK). For anonymity, all study participants were assigned an individual study-specific identification code and all laboratory analysis was carried out blinded from any other data.

### 2.2. Parasitological survey sample sites

**Fig 1:**
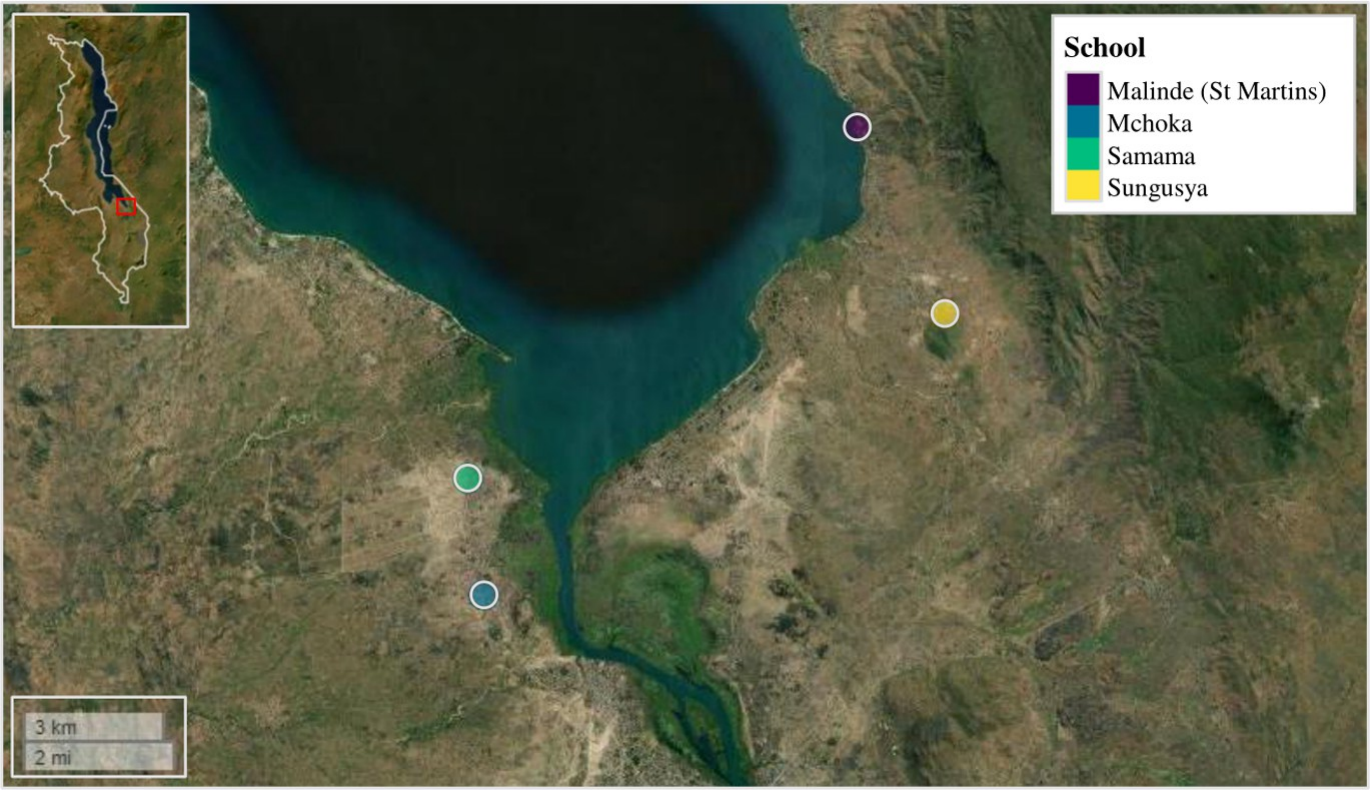
Samama, Mchoka, Sungusya and Malinde (St. Martins) primary schools, situated along the southern shoreline of Lake Malawi, Mangochi District, Malawi. Malawi’s country border can be seen within figure inset (upper left corner). Within figure inset, study area is highlighted by red box. Figure generated using the ‘mapview’ package version 2.10.0 [22] within R Studio version 2021.09.0, build 351 (Posit, USA) [23].

Four primary schools situated along the southern shoreline of Lake Malawi, Mangochi District, Malawi, were selected for parasitological surveillance. Primary schools are routinely targeted for schistosomiasis surveillance in endemic areas as the highest burdens of schistosomiasis infection are typically observed in school-aged children and young adolescents. Two schools on the western side of the lake and two on the eastern side of the lake were selected based on their close proximity to the lake’s shoreline and previously identified prevalence’s of intestinal and/or urogenital schistosomiasis in school-aged children: suggesting frequent community lake water contact [10,18]. These were: Samama School (latitude: -14.417465°; longitude: 35.217580°); Mchoka School (latitude: -14.439481°; longitude: 35.220644°); Sungusya School (latitude: -14.386472°; longitude: 35.311398°) and Malinde (St Martins) School (latitude: -14.351401°; longitude: 35.294435°), (Fig 1). In total, 362 school-aged children (range: 6–16 years; mean age: 10.6 years) were surveyed across all four primary schools (Samama School *n* = 121; Mchoka School *n* = 121; Sungusya School *n* = 60 and Malinde (St Martins) School *n* = 61). The number of study participants was weighted more towards schools situated on the western side of the lake based on previous parasitological surveys and anticipated prevalence of schistosomiasis infection [10,18].

### 2.3. Faecal and urine sample provision and initial diagnostic procedures

A single faecal and urine sample were provided by 313 (86.2%) study participants across all four primary schools between 10:00 am and 14:00 pm to coincide with *Schistosoma* spp. egg excretion patterns as described previously [15], (Samama School *n* = 108; Mchoka School *n* = 105; Sungusya School *n* = 53 and Malinde (St Martins) School *n* = 47), (age range: 6–16 years; mean age: 10.8 years). A proportion of each faecal sample was used for Kato-Katz faecal-egg microscopy as described below. In addition, approximately 1 gram of faecal material was removed from the inner contents of each stool sample (rather than the outer edge), filtered through a standard 212 μM gauge filter, and stored within a labelled 2 mL screwcap tube containing 1 mL 100% ethanol for preservation. Nitrile gloves were worn at all times during handling of faecal and urine material. All ethanol preserved faecal material was then transported to the LSTM under ambient conditions for DNA extraction and molecular analysis. A proportion of urine material from each urine sample was used for urine-egg microscopy (10 ml) and POC-CCA testing, as also described below. All initial diagnostic testing and sample preservation was carried out no longer than nine hours post sample provision.

#### 2.3.1. Kato-Katz faecal-egg microscopy

Kato-Katz faecal-egg microscopy was used to diagnose intestinal schistosomiasis and quantify expelled *S*. *mansoni* ova within stool samples according to a standard protocol [7]. Kato-Katz smears were prepared in duplicate upon one single labelled glass slide. Each slide, containing both smears, was then read by two independent trained microscopists. Slides were deemed negative for infection and scored as ‘0’ if no *S*. *mansoni* ova were observed. If *S*. *mansoni* ova were observed, slides were deemed as positive for infection and the total number of eggs observed in both smears was multiplied by 24. This value was then divided by two to quantify the number of eggs per gram (epg) of faeces. The mean epg of faeces across both readings was then calculated to determine the epg of faeces per participant. Positive Kato-Katz outcomes were scored according to *S*. *mansoni* epg of faeces using the following categories: light infection (1–99 ova per 1 g faeces): ‘1’; moderate infection (100–399 ova per 1 g faeces): ‘2’; heavy infection (≥ 400 ova per 1 g faeces): ‘3’ [24].

#### 2.3.2. Point-of-care circulating cathodic antigen (POC-CCA) RDT (ICT International, South Africa)

Circulating cathodic antigen lateral-flow RDTs (POC-CCA) were also used to diagnose infection with *S*. *mansoni* according to manufacturer’s instructions. This highly portable lateral-flow cassette immunoassay is used to detect *S*. *mansoni* circulating cathodic antigens excreted within urine samples [14]. POC-CCA assays that did not present a visible test band were deemed as negative for infection and scored as ‘0’, whereas those that presented a visible test line were deemed as positive for infection and scored according to band strength; light infection (presenting a faint or ‘trace’ band): 1; moderate infection (presenting a moderate band): ‘2’; heavy infection (presenting a strong band): ‘3’. As it is not currently clear whether ‘trace’ bands represent an active infection, or are in fact false-positive diagnostic outcomes, all data analyses were performed considering ‘trace’ as positive and then as repeated considering ‘trace’ as negative [25,26]. Any POC-CCA assays that did not present a visible control line were repeated.

#### 2.3.3. Urine-egg microscopy

Urine-egg microscopy was used to diagnose urogenital schistosomiasis and quantify expelled *S*. *haematobium* ova according to a standard protocol [15]. Urine samples were gently agitated by hand to resuspend ova and 10 ml was drawn using a syringe. This 10 ml aliquot was then filtered using a 20 μM gauge filter, which was then placed onto a labelled glass slide using forceps for microscopy. Each preparation was read by a trained microscopist. In total, 309 of 313 (98.7%) urine samples were analysed as four were inadequate in volume (Samama School *n* = 106; Mchoka School *n* = 103; Sungusya School *n* = 53 and Malinde (St Martins) School *n* = 47). Urine-egg microscopy preparations were deemed negative for infection and scored as ‘0’ if no *S*. *haematobium* ova were observed and deemed positive if *S*. *haematobium* ova were observed. Positive urine-egg microscopy preparations were scored according to the number of *S*. *haematobium* eggs observed per 10 mL urine using the following categories: light infection (1–9 ova per 10 mL urine): ‘1’; moderate infection (10–49 ova per 10 mL urine): ‘2’; heavy infection (> 50 ova per 10 mL urine): ‘3’. Once 50 *S*. *haematobium* eggs were counted, the urine-egg microscopy preparation was scored as heavy infection, and no further counting took place.

### 2.4. Collection of *Schistosoma* spp. miracidia hatched from faecal samples

Approximately five grams of faecal material provided by 10 participants at Samama school deemed positive for infection with *S*. *mansoni* by POC-CCA (moderate or heavy infections) was pooled and filtered using a Pitchford-Visser funnel according to a standard miracidium hatching technique (MHT) protocol [27]. This was then repeated using faecal material provided by 10 participants at Mchoka school. This protocol is used to isolate *Schistosoma* ova from faecal and urine material. Isolated ova were deposited into fresh petri dishes containing clean, bottled water. Petri dishes were then exposed to a combination of natural and artificial light at ambient temperature for approximately three hours to induce miracidial hatching. Petri dishes were examined under a dissecting microscope to check for any hatched miracidia. Hatched and motile miracidia were then individually captured using a micropipette (2.5 μl) and deposited/stored onto Whatman FTA cards for DNA preservation (GE Healthcare Life Sciences, UK). FTA cards were stored according to manufacturer’s instructions and transported to the London Natural History Museum (NHM) under ambient conditions for molecular analyses.

### 2.5. Molecular diagnosis of intestinal schistosomiasis

DNA extracted from faecal samples was analysed by real-time PCR using a two-step approach. All samples were initially screened for the presence of *Schistosoma* spp. DNA using a routine genus-specific diagnostic real-time PCR assay according to a standard protocol [28]. All samples positive for the presence of *Schistosoma* spp. DNA were then screened for the presence of *S*. *mansoni* and *S*. *haematobium* DNA specifically, using a secondary species-specific duplex real-time PCR. All real-time PCR assay plates were prepared using a Myra liquid handling system (Bio Molecular Systems (BMS), UK) and all real-time PCR assays were carried out using a Magnetic Induction Cycler (MIC) PCR thermocycler (Bio Molecular Systems (BMS), UK).

#### 2.5.1. DNA extraction: Ethanol preserved faecal samples

DNA was isolated from approximately 0.2 g of each ethanol preserved faecal sample using the QIAamp DNA mini kit (QIAGEN, Germany) according to manufacturer’s instructions with minor revisions including a bead-beating step using a MagNA Lyser cell disrupter [Roche Applied Science, Germany], (30 seconds; 3,000 RPM) as described previously [29]. During DNA extraction, phocine herpes virus 1 (PhHV-1) was added to the ATL buffer and proteinase K mix (2 μl PhHV-1 per sample) to act as an internal DNA extraction control [30]. DNA extractions were performed in batches of 23 faecal samples. Each batch also included one DNA extraction-negative control sample that was subjected to the same DNA extraction protocol but did not contain any faecal material.

#### 2.5.2. Genus-specific *Schistosoma* spp. ITS2 rDNA real-time PCR

All faecal DNA samples were tested using a routine duplex genus-specific diagnostic real-time PCR to target and amplify a 77 bp fragment of the *Schistosoma* spp. nuclear internal transcribed spacer 2 (ITS2) ribosomal DNA (rDNA) region, as well as an 89 bp fragment of the PhHV-1 glycoprotein B gene which acts as a DNA extraction and PCR internal positive control. Details of primer/probe sequences, reaction mix used, and PCR conditions are described in Table A, Table B, and Table C in S1 File, respectively. All ITS2 real-time PCRs included one positive control using *S*. *mansoni* template DNA extracted from one *S*. *mansoni* adult male worm provided by the Schistosome and Snail Resource (SSR) based across both the NHM and the London School of Hygiene and Tropical Medicine (LSHTM), UK [31]. ITS2 real-time PCRs also included one DNA extraction-negative control using template DNA extracted from a DNA extraction-negative sample that did not contain any faecal material and one no-template negative control using ddH_2_O in place of template DNA. Any samples that did not amplify PhHV-1 DNA were tested again within a secondary repeat screen. Any samples that did not amplify PhHV-1 DNA during either PCR were considered to have failed DNA extraction and so were not included in any further analysis. An ITS2 cycle threshold (Ct) value of ≤ 39 was deemed positive for the presence of *Schistosoma* spp. DNA, whereas an ITS2 Ct value of ≥ 40 was deemed negative for the presence of *Schistosoma* spp. DNA. For quality assurance, 10% of *Schistosoma* spp. positive and 10% of *Schistosoma* spp. negative samples were retested.

#### 2.5.3. Species-specific *S*. *mansoni* and *S*. *haematobium* mitochondrial 16*s* rDNA real-time PCR

All faecal DNA samples positive for the presence of *Schistosoma* spp. DNA during the initial ITS2 real-time PCR were then tested using a duplex species-specific real-time PCR targeting *S*. *mansoni-* and *S*. *haematobium*-specific mitochondrial 16S rDNA loci (104 bp and 143 bp in length, respectively), [32]. Details of primer/probe sequences, reaction mix used, and PCR conditions are described in Table D, Table E, and Table, F in S1 File, respectively. All 16S real-time PCRs included one positive control using *S*. *mansoni* template DNA as described above, one positive control using *S*. *haematobium* template DNA extracted from one *S*. *haematobium* adult worm provided by the Schistosome Collections at the Natural History Museum (SCAN) repository [33], and one positive control using a 1:1 mix of both *S*. *mansoni* and *S*. *haematobium* adult worm DNA. All 16S real-time PCRs also included one no-template negative control using ddH_2_O in place of template DNA. For both *S*. *mansoni* and *S*. *haematobium* loci, a cycle threshold (Ct) value of ≤ 39 was deemed positive for infection whereas a Ct value of ≥ 40 was deemed negative for infection. For quality assurance, 10% of *S*. *mansoni*-positive only, 10% *S*. *haematobium*-positive only, 10% of *S*. *mansoni-* and *S*. *haematobium*-positive and 10% of samples that failed to amplify either *S*. *mansoni* or *S*. *haematobium* loci were retested.

2.5.3.1 Species-specific *S*. *mansoni* and *S*. *haematobium* mitochondrial *cox*1 rDNA end-point PCR and Sanger sequencing. To confirm that the species-specific 16S real-time PCR was detecting and amplifying *S*. *mansoni* and *S*. *haematobium* DNA, a multiplex end-point PCR was carried out targeting the mitochondrial *cox*1 rDNA loci of different *Schistosoma* species [34]. This species-specific PCR uses one universal (genus-specific) forward primer and four distinct reverse primers specific to *S*. *mansoni*, *S*. *haematobium*, *Schistosoma mattheei* and *Schistosoma bovis*, to detect and amplify four *cox*1 regions that differ in length according to *Schistosoma* species (375 bp, 543 bp, 362 bp and 306 bp, respectively) [34]. This was done using 20% of samples deemed positive for *S*. *mansoni* only, 20% of samples deemed positive for *S*. *haematobium* only, 20% of samples deemed positive for both *S*. *mansoni* and *S*. *haematobium*, and 20% of samples deemed negative for both *S*. *mansoni* and *S*. *haematobium*, by 16S real-time PCR. Details of all primer sequences, reaction mixes used, and PCR conditions are described in Table G, Table H, and Table I in S1 File, respectively.

*Cox*1 PCRs included four positive controls using *S*. *mansoni* and *S*. *haematobium* DNA as described above as well as *S*. *mattheei* and *S*. *bovis* DNA extracted from one adult male worm of both species provided by SCAN. *Cox*1 PCRs also included one no-template negative control using ddH_2_O in place of template DNA. Amplicons were visualised by running 7.5 μl PCR product mixed with 2 μl 5x loading buffer blue [Bioline, UK] stained with GelRed on a 3% agarose gel. Five randomly selected *S*. *mansoni* infection only and five randomly selected *S*. *haematobium* infection only *cox*1 PCR products were purified using the QIAquick PCR purification kit [QIAGEN, UK] according to manufacturer’s instructions and Sanger sequenced in the reverse direction using a dilution of Sman_RV (*S*. *mansoni-*specific) and Sh_RV (*S*. *haematobium-*specific) reverse primers. Sequence data was visualised, trimmed, and edited as needed using Geneious Prime version 2023.01 [Biomatters, LTD] and identified using the Basic Local Alignment Search Tool (BLAST) algorithm within the NCBI database [35].

### 2.6. Statistical analysis

All statistical analysis was carried out in RStudio version 2024.04.2 build 764 (R version 4.4.1), [23]. Sensitivity, specificity, positive predictive and negative predictive values were calculated using the ‘epiR’ package version 2.0.39 [36]. All regression analysis and analysis of variance (ANOVA)/ post hoc Tukey’s honestly significant difference (HSD) for multiple comparisons testing was performed using the ‘ R stats’ package version 4.5.0 [23].

#### 2.6.1. Diagnostic outcome of Kato-Katz faecal-egg microscopy and POC-CCA compared to real-time PCR

The diagnostic outcome (positive/negative) of Kato-Katz faecal-egg microscopy and POC-CCA was compared to that of real-time PCR (derived from positive *S*. *mansoni* 16S real-time PCR outcomes, negative *S*. *mansoni* 16S real-time PCR outcomes, and negative *Schistosoma* spp. ITS2 real-time PCR outcomes and considered here as the reference standard assay). To do this, sensitivity, specificity, positive predictive and negative predictive values were calculated. For POC-CCA, these were calculated considering ‘trace’ results as a positive diagnostic outcome as well as considering ‘trace’ results as a negative diagnostic outcome [25,26]. Linear regression was then used to assess if positive POC-CCA band strength scores (considered categorical data as successive categories are not equally spread) significantly predicted *S*. *mansoni* faecal 16S real-time PCR Ct values (derived from *S*. *mansoni* 16S real-time PCR positive results and considered a proxy of *S*. *mansoni* infection intensity) when compared to negative *S*. *mansoni* POC-CCA outcomes. Following this, a one-way analysis of variance (ANOVA) was carried out to assess if there were any statistically significant differences between any *S*. *mansoni* POC-CCA band strength scores when compared to *S*. *mansoni* 16S real-time PCR Ct values.

#### 2.6.2. Measuring any associations between *S*. *haematobium* faecal 16S real-time PCR and urine-egg microscopy

The diagnostic outcome (positive/negative) of the *S*. *haematobium* faecal 16S real-time PCR was compared to that of urine-egg microscopy (considered here as the reference standard assay). To do this, sensitivity, specificity, positive predictive and negative predictive values were calculated. Linear regression was then used to assess if *S*. *haematobium* urine-egg output per 10 mL urine (considered categorical data as successive categories are not equally spread) significantly predicted *S*. *haematobium* faecal 16S real-time PCR Ct values (derived from *S*. *haematobium* faecal 16S real-time PCR positive results and considered a potential proxy of *S*. *haematobium* infection intensity). Following this, a one-way ANOVA with post hoc Tukey’s HSD test for multiple comparisons was carried out to assess if mean differences between *S*. *haematobium* urine-egg output categories and *S*. *haematobium* faecal 16S real-time PCR Ct values were significantly different.

### 2.7. Molecular characterisation of *Schistosoma* miracidia

To genotype the *Schistosoma* spp. miracidia, a multi-locus approach targeting both mitochondrial (maternally inherited) and nuclear (inherited equally from both parents) DNA to identify single- or multi-species ancestry was used [16,17].

#### 2.7.1. DNA extraction: FTA preserved miracidia

DNA was isolated from all FTA preserved *Schistosoma* spp. miracidia according to a standard protocol [37]. Prior to DNA extraction, miracidia underwent a purification step that involved completely submerging each punched FTA spot in 200 μl QIAcard FTA wash buffer (QIAGEN, UK) immediately after punching from Whatman FTA cards. Submerged punches were then incubated at room temperature for five minutes. The FTA purification buffer was then removed and 200 μl TE x1 was added, again ensuring all punches were completely submerged. Submerged punches were again incubated at room temperature for five minutes and the TE x1 was removed.

#### 2.7.2. Mitochondrial *cox*1 and nuclear ITS genotyping

To genotype miracidia, PCR was used to amplify a 956 bp region of the *Schistosoma* spp. mitochondrial *cox*1 gene according to a standard protocol [38]. Details of all primer sequences, reaction mixes used, and PCR conditions are described in Table J, Table K, and Table L in S1 File, respectively. *Cox*1 PCRs included a positive control using *S*. *mansoni* DNA collectively isolated from three *S*. *mansoni* adult worms provided by the SSR and a negative control using ddH_2_O in place of template DNA. Amplicons were visualised by running 4 μl of PCR products mixed with 1.5 μl 5x loading buffer blue [Bioline, UK] stained with GelRed on a 1% agarose gel. *Schistosoma* spp. *cox*1 PCR products were purified as described above and Sanger sequenced in the reverse direction using a dilution of the Schisto_3’ reverse primer. Sequence data was visualised, trimmed, edited, and identified as described above.

PCR was then used to amplify the complete *Schistosoma* spp. nuclear internal transcribed spacer (ITS) region (~1005 bp) according to a standard protocol [38]. Details of all primer sequences, reaction mixes used, and PCR conditions are described in Table M, Table N, and Table O in S1 File, respectfully. ITS PCRs included one positive and one negative control as with *Schistosoma* spp. *cox*1 PCRs. Amplicons were visualised as described above. *Schistosoma* spp. ITS PCR products were purified as described above and Sanger sequenced in the reverse direction using a dilution of the ETTS1 reverse primer. Sequence data was visualised, trimmed, edited, and identified as described above.

2.7.2.1 Examination of *S*. *mansoni* and *S*. *haematobium* miracidia nuclear ITS chromatograms for evidence of mixed-species ancestry. All ITS chromatograms were examined for double-peaking at heterozygous species-specific single nucleotide polymorphism (SNP) sites between reference *S*. *mansoni*, *S*. *rodhaini*, *S*. *haematobium*, *S*. *mattheei* and *S*. *bovis* ITS sequence data to check for any evidence of mixed-species ancestry [38] (GenBank Accession numbers: MF776590, AY157202, MT580959, OR062311 and FJ588862, respectively). To do this, reverse complement forward sequences to all generated Mangochi District, Malawi *S*. *mansoni* ITS reverse sequences were generated within Geneious Prime and aligned to all downloaded *Schistosoma* spp. ITS sequence data using a MAFFT alignment (default MAFFT parameter settings). The MAFFT alignment was then examined for chromatogram double-peaking at any SNP sites.

#### 2.7.3. Identification of *S*. *mansoni cox*1 lineage groups

To identify which previously defined *S*. *mansoni cox*1 lineage group (I–V) Mangochi District *S*. *mansoni* miracidia were most closely related to [16], *cox*1 sequence data (all unique *S*. *mansoni cox*1 haplotypes) from both lineage groups I and II (randomly selected whilst representing all available sampling locations) were downloaded from the GenBank repository. This represented 35.4% and 46% of all available haplotype sequences, respectively, (see S2 File, Table A). In addition, all available unique haplotype *S*. *mansoni cox*1 sequence data from lineage groups III, IV and V were also downloaded (*n* = 15, *n* = 18 and *n* = 7, respectively; see S2 File, Table A). Each lineage group represents the following geographical topologies: Group I: Far west Africa (Senegal and Mali), Brazil, Egypt, Saudi Arabia, and Oman; Group II: East Africa (Kenya, Uganda, Tanzania, and Zambia); Group III: central west Africa (Cameroon) and Niger; Group IV: Zambia and Coastal Kenya and Group V: Zambia [16].

All Mangochi District, Malawi, *S*. *mansoni cox*1 sequence data (*n* = 24 sequences) were aligned to all downloaded *S*. *mansoni cox*1 sequence data using a multiple alignment with fast fourier transform (MAFFT) alignment, (default MAFFT parameter settings) in Geneious Prime. The MAFFT alignment was visualised, trimmed (to ensure uniform ends across all sequences) and examined as described above. The alignment was then exported from Geneious Prime in Nexus file format and imported into PopART version 1.7 [39]. Within PopART, a Templeton Crandall and Sing (TCS) network [40] was generated to allow examination of haplotype group structuring. The MAFFT alignment was then uploaded into DNAsp version 6.12.03 [41], where all downloaded *S*. *mansoni cox*1 data was grouped according to their previously defined lineage group I-V [16]. Mangochi District, Malawi, *S*. *mansoni cox*1 haplotypes were then also grouped according to which *S*. *mansoni cox*1 lineage group they most closely clustered to. The net evolutionary divergence (*D*_*xy*_), defined as the average number of SNPs across haplotypes between lineage groups, between lineage groups I–V, Mal-II, and Mal-IV, was then calculated using a Juke-Cantor correction model within DNAsp [16]. Following this, the generated distance matrix was used to construct an unrooted neighbour joining (NJ) phylogenetic tree using the Trex-Online phylogenetic tree inference tool [42] to visualise net evolutionary divergence scores between all five *S*. *mansoni cox*1 lineage groups as well as between Mal-II, and Mal-IV groups.

## 3. Results

### 3.1. Diagnosis of intestinal and urogenital schistosomiasis

All diagnostic data concerning intestinal and urogenital schistosomiasis are summarised in Tables 1 and 2, respectively.

**Table 1:** Summary of all diagnostic data concerning intestinal schistosomiasis.

School	Kato-Katz faecal microscopy		POC-CCA*		*Schistosoma* spp. ITS2 real-time PCR*		*S*. *mansoni* 16S real-time PCR*	
	Positive % (Total screened)	Mean epg*	‘Trace’ +* % (Total screened)	‘Trace’ -* % (Total screened)	Positive % (Total screened)	Mean Ct*	Positive %	Mean Ct*
**Samama**	5.7% (6 of 105)	26.4	41.9% (44 of 105)	27.6% (29 of 105)	67.6.% (71 of 105)	25.5	55.2% (58 of 105)	29.8
**Mchoka**	2.9% (3 of 102)	31.2	51% (52 of 102)	41.2% (42 of 102)	59.8% (61 of 102)	29.5	37.2% (38 of 102)	31.8
**Sungusya**	1.9% (1 of 52)	12	9.6% (5 of 52)	7.7% (4 of 52)	11.5% (6 of 52)	31.6	14% (6 of 43)	33.3
**Malinde** **(St Martins)**	0% (0 of 46)	0	28.3% (13 of 46)	10.9% (5 of 46)	23.9% (11 of 46)	30.9	12.8% (5 of 39)	34.7
**All**	3.3% (10 of 305)	26.4	37.4% (114 of 305)	26.2% (80 of 305)	48.9% (149 of 305)	28	37% (107 of 289)	31

* *Where*:

Mean epg: Mean number of *S*. *mansoni* eggs per gram of faeces identified during Kato-Katz faecal-egg microscopy

POC-CCA: Circulating cathodic antigen rapid diagnostic test

‘Trace +’: POC-CCA ‘trace’ results considered a positive diagnostic outcome

‘Trace–‘: POC-CCA ‘trace’ results considered a negative diagnostic outcome

ITS2 real-time PCR: *Schistosoma* spp. genus-specific real-time PCR targeting ITS2 locus

*S*. *mansoni* 16S real-time PCR: *S*. *mansoni*-specific real-time PCR targeting 16S locus

Mean Ct: Mean real-time PCR cycle threshold value

**Table 2:** Summary of all diagnostic data concerning urogenital schistosomiasis.

School	Urine-egg microscopy		*Schistosoma* spp. faecal ITS2 real-time PCR *		*S*. *haematobium* faecal 16S real-time PCR*	
	Positive % (Total screened)	Proportion of heavy infections* %	Positive % (Total screened)	Mean Ct*	Positive %	Mean Ct*
**Samama**	76.2% (80 of 105)	47.5% (38 of 80)	67.6.% (71 of 105)	25.5	39% (41 of 105)	29
**Mchoka**	37.3% (38 of 102)	44.7% (17 of 38)	59.8% (61 of 102)	29.5	28.4% (29 of 102)	28.5
**Sungusya**	17.3% (9 of 52)	44.4% (4 of 9)	11.5% (6 of 52)	31.6	11.5% (5 of 43)	33
**Malinde** **(St Martins)**	13% (6 of 46)	33.4% (2 of 6)	23.9% (11 of 46)	30.9	2.6% (1 of 39)	33.9
**All**	43.6% (133 of 305)	45.9% (61 of 133)	48.9% (149 of 305)	28	26.3% (76 of 289)	28.8

* *Where*:

Proportion of heavy infections: Proportion of participants positive for urogenital schistosomiasis by urine-egg microscopy harbouring heavy *S*. *haematobium* infections (> 50 eggs per 10 mL urine)

ITS2 real-time PCR: *Schistosoma* spp. genus-specific faecal real-time PCR targeting ITS2 locus

*S*. *haematobium* faecal 16S real-time PCR: *S*. *haematobium*-specific faecal real-time PCR targeting 16S locus

Mean Ct: Mean real-time PCR cycle threshold value

#### 3.1.1. Kato-Katz faecal-egg microscopy

A total of 10 participants (3.3%) were positive for *S*. *mansoni* infection using Kato-Katz faecal microscopy, all of which were light infections (25.4 mean epg (range 12–48 epg) across all 10 participants). No ectopic *S*. *haematobium* eggs were identified in any Kato-Katz preparations.

#### 3.1.2. POC-CCA

When POC-CCA ‘trace’ results were considered positive, 114 participants (37.4%) were positive for *S*. *mansoni* infection. Of these, the POC-CCA assay suggested that 30% (11.8% of all participants) had a ‘trace’ infection; 65% (24.3% of all participants) had a moderate intensity infection and 5% (1.3% of all participants) had a heavy intensity infection. When POC-CCA ‘trace’ results were considered negative, 80 participants (26.2%) were positive for *S*. *mansoni* infection. All POC-CCA assays gave a positive control line.

#### 3.1.3. Urine-egg microscopy

A total of 133 participants (43.6%) were positive for *S*. *haematobium* infection using urine-egg microscopy. Of these, 27% (11.8% of all participants) were light intensity infections; 27% (11.8% of all participants) were moderate intensity infections and 45.9 (20% of all participants) were heavy intensity infections. Lateral spined *S*. *mansoni-*shaped eggs were observed in the urine of six (1.9%) participants.

#### 3.1.4. Real-time PCR detection of *Schistosoma* spp. ITS2 nuclear DNA and *S*. *mansoni*- and *S*. *haematobium*-specific mitochondrial 16S DNA

DNA was successfully extracted from 310 of all 313 (99%) ethanol preserved faecal samples as three faecal samples were too low in volume. Of the 310 samples screened, five failed to amplify the PhHV-1 internal DNA extraction control in both initial and repeat screens and were therefore omitted from further analysis. Of the remaining 305 samples, 149 (48.9%) were positive for *Schistosoma* spp. infection using the ITS real-time PCR (s 1 and 2).

Of the 149 samples positive for the presence of *Schistosoma* spp. DNA using the genus-specific ITS2 real-time PCR, only the *S*. *mansoni-*specific 16S locus was amplified in 53 (35.6%) of these (17.4% of all participants), only the *S*. *haematobium-*specific 16S locus was amplified in 26 (15%) of these (9% of all participants), and both *S*. *mansoni-* and *S*. *haematobium-*specific 16S loci were amplified in 54 (37%) of these (18% of all participants). Neither *S*. *mansoni-* nor *S*. *haematobium-*specific 16S loci were amplified in 16 (10.7%) samples (nine of these were from Sungusya School and seven of these were from Malinde (St Martins) School; the mean ITS2 real-time PCR Ct value of these 16 samples was Ct 35.7, whereas the mean ITS2 real-time PCR Ct value of all ITS2 positive samples was 28). In total, 107 samples were positive for the presence of *S*. *mansoni* DNA using the species-specific 16S real-time PCR. The overall prevalence of *S*. *mansoni* infection was therefore 37% using the two-step real-time PCR approach (1). For all real-time PCR assays, all samples retested for quality assurance gave the same diagnostic outcome as the initial screen with positive samples giving a Ct value within ± Ct 3 of that given during the initial screen.

The diagnostic outcome of the *Schistosoma* species-specific *cox*1 PCR matched that of the 16S real-time PCR in all screened samples bar one sample that had amplified only the *S*. *mansoni* 16S locus during real-time PCR screening but had failed to amplify the *S*. *mansoni cox*1 locus during the *cox*1 PCR. The *cox*1 PCR was repeated using this sample, but again failed to amplify the *S*. *mansoni cox*1 locus. Neither *S*. *mattheei* nor *S*. *bovis cox*1 loci were amplified in any samples. All five *S*. *mansoni* and all five *S*. *haematobium cox*1 PCR amplicons were confirmed as *S*. *mansoni* and *S*. *haematobium*, respectively, using BLAST analysis. All *S*. *mansoni* and *S*. *haematobium cox*1 sequence data was uploaded to the GenBank repository (Accession numbers: PP531045—PP531049 and PP531370 –PP531374, respectively).

### 3.2. Statistical analysis

#### 3.2.1. Diagnostic outcome of Kato-Katz faecal-egg microscopy and POC-CCA compared to real-time PCR

Sensitivity, specificity, positive predictive and negative predictive values of Kato-Katz faecal-egg microscopy and POC-CCA compared to real-time PCR are summarised in Table 3.

**Table 3:** Sensitivity, specificity, positive predictive and negative predictive values of Kato-Katz faecal-egg microscopy and POC-CCA compared to *S*. *mansoni* 16S real-time PCR.

Reference standard (Total screened)	Index test	Sensitivity	Specificity	PPV*	NPV*
***S*. *mansoni* 16S** real-time PCR* (*n* = 289)	**Kato-Katz** **faecal microscopy** (*n* = 289)	9% (95% CI: 4–16)	100% (95% CI: 98–100)	100% (95% CI: 69–100)	66% (95% CI: 60–71)
	POC-CCA (‘trace’ +)* (*n* = 289)	73% (95% CI: 64–81)	81% (95% CI: 76–86)	66% (95% CI: 57–74)	86% (95% CI: 81–90)
	POC-CCA (‘trace’ -)* (*n* = 289)	56% (95% CI: 46–65)	90% (95% CI: 85–93)	72% (95% CI: 61–82)	80% (95% CI: 75–85)

* *Where*:

*S*. *mansoni* 16S real-time PCR: *S*. *mansoni*-specific faecal real-time PCR targeting 16S locus

POC-CCA ‘trace +’: POC-CCA ‘trace’ results considered positive

POC-CCA ‘trace–‘: POC-CCA ‘trace’ results considered negative

PPV: Positive predictive value

NPV: Negative predictive value

**Fig 2:**
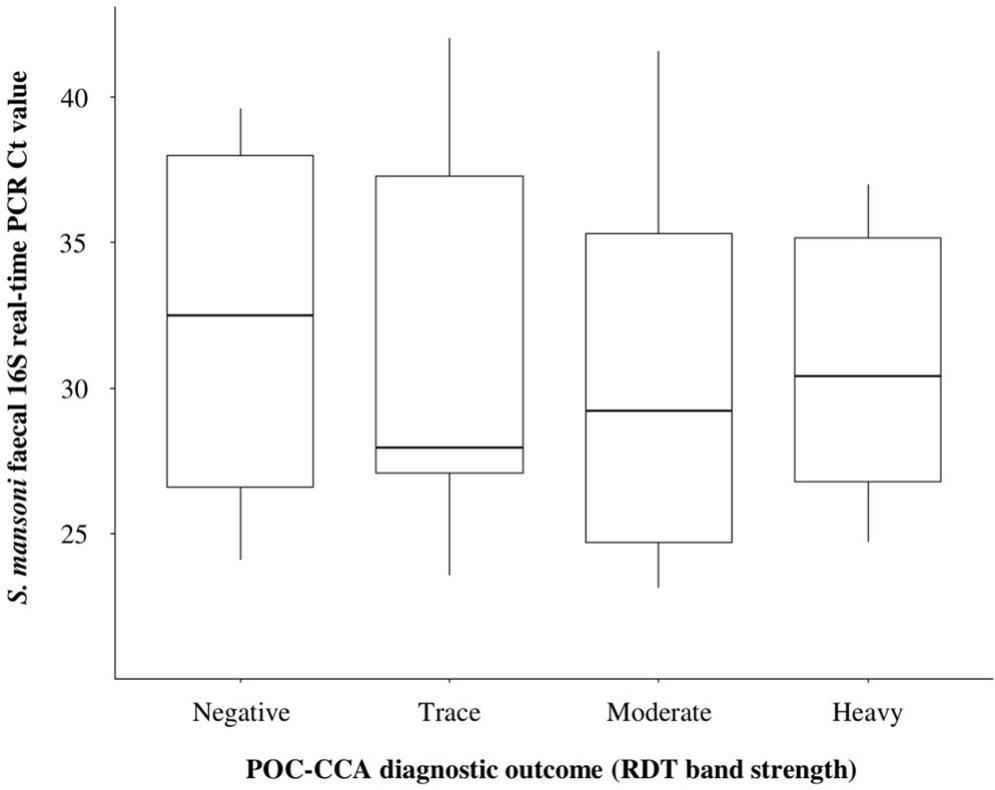
Boxplot showing the median differences between *S*. *mansoni* POC-CCA diagnostic outcome categories and *S*. *mansoni* faecal 16S real-time PCR Ct values. For each *S*. *mansoni* POC-CCA diagnostic outcome category, *S*. *mansoni* faecal 16S real-time PCR Ct value medians, interquartile ranges and ranges are shown. Plot generated using ‘ggplot2’ package version 3.4.0 3.4.0 [43].

Of the 34 POC-CCA assays that gave a ‘trace’ positive outcome, 29 (85.3%) of these were also ITS2 real-time PCR positive. Of these 29, 19 (65.5%) were *S*. *mansoni* 16S real-time PCR positive whereas the remaining 10 samples (34.5%) were *S*. *mansoni* 16S real-time PCR negative. The average Ct value of these 19 *S*. *mansoni* 16S real-time PCR positive samples was 31.7. By comparison, the average Ct value of *S*. *mansoni* 16S real-time PCR positive samples that were deemed moderate and heavy intensity infections by POC-CCA was 30.3, and so there was little comparable difference between mean *S*. *mansoni* 16S real-time PCR Ct values deemed ‘trace’ infection by POC-CCA, and mean *S*. *mansoni* 16S real-time PCR Ct values deemed moderate and heavy intensity infections by POC-CCA. Additionally, 30 samples that were POC-CCA negative were *S*. *mansoni* 16S real-time PCR positive. The average Ct value of these 30 *S*. *mansoni* 16S real-time PCR positive samples was 31.9. By comparison, the average Ct value of *S*. *mansoni* 16S real-time PCR positive samples across all POC-CCA positive samples was 30.6. and so there was little comparable difference between mean *S*. *mansoni* 16S real-time PCR values deemed negative by POC-CCA and mean *S*. *mansoni* 16S real-time PCR Ct values deemed positive by POC-CCA. Overall, there were 27 POC-CCA positive samples that were *S*. *mansoni* 16S real-time PCR negative. Of these, 14 (52%) were positive for *S*. *haematobium* infection by urine-egg microscopy, with five categorised as heavy intensity infection, three categories as moderate intensity infection, and six categories as light infection. The boxplot comparing POC-CCA band strength and *S*. *mansoni* 16S real-time PCR values can be seen in Fig 2.

For all POC-CCA band strength scores, including ‘0’ (negative), there was a large *S*. *mansoni* 16S real-time PCR Ct value range and interquartile range. The POC-CCA band strength that had the lowest *S*. *mansoni* 16S real-time PCR Ct value was POC-CCA score ‘1’ (‘trace’ positive). The fitted linear model was statistically insignificant (R^2^ = 0.07, F = 0.6, p = 0.62) and so no POC-CCA band strengths were found to significantly predict *S*. *mansoni* 16S real-time PCR Ct values when compared to a negative POC-CCA outcomes (Table 4). The one-way ANOVA revealed that there were also no statistically significant differences between any POC-CCA band strengths and *S*. *mansoni* 16S real-time PCR Ct values (F = 0.601, p = 0.62).

**Table 4:** Linear regression correlation coefficient estimates comparing *S*. *mansoni* POC-CCA diagnostic outcome categories and *S*. *mansoni* faecal 16S real-time PCR Ct values.

POC-CCA outcome	Mean difference from intercept (Ct 31.89), (± 95% CI*)	Standard Error	t value	p value
Negative (Intercept)	31.89 (29.76–34.01)	/	/	/
Trace	-0.23 (-3.63–3.17)	1.71	-0.14	0.89^†^
Moderate	-1.63 (-4.27–1.01)	1.33	-1.22	0.24^†^
Heavy	-1.08 (-6.29–4. 12)	2.62	-0.41	0.68^†^

Where:

*95% Confidence Intervals

^*‡*^: Statistically significant

^†^: Not statistically significant

#### 3.2.2. Measuring any association between *S*. *haematobium* faecal 16S real-time PCR Ct values and urine-egg microscopy

**Fig 3:**
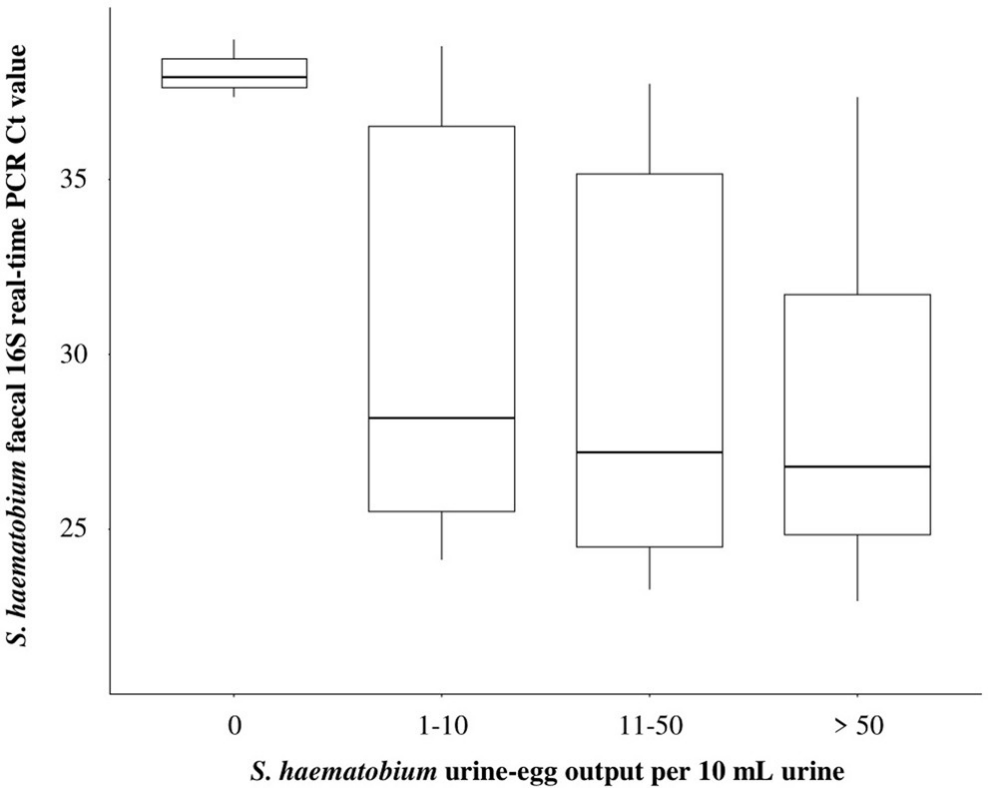
Boxplot showing the median differences between *S*. *haematobium* urine-egg output categories (*S*. *haematobium* eggs per 10 mL urine) and *S*. *haematobium* faecal 16S real-time PCR Ct values. For each *S*. *haematobium* urine-egg output category, *S*. *haematobium* faecal 16S real-time PCR Ct value medians, interquartile ranges and ranges are shown. Plot generated using ‘ggplot2’ package version 3.4.0 3.4.0 [43].

When comparing the *S*. *haematobium* faecal 16S real-time PCR outcome against urine-egg microscopy, the *S*. *haematobium* 16S real-time PCR had a sensitivity of 78% (95% CI: 68–86), a specificity of 89% (95% CI: 80–95), a positive predictive value of 90% (95% CI: 91–96) and a negative predictive value of 76% (95% CI: 66–85). Of the 20 individuals positive for *S*. *haematobium* infection by urine-egg microscopy but negative by *S*. *haematobium* faecal 16S real-time PCR, only three (15%) were heavy intensity infections (4% of all participants with heavy intensity *S*. *haematobium* infections). In addition, eight (40%) of these were moderate intensity infections (20% of all participants with moderate intensity *S*. *haematobium* infections) and nine (45%) of these were light intensity infections (21% of all participants with light intensity *S*. *haematobium* infections). A boxplot comparing *S*. *haematobium* urine-egg output categories and *S*. *haematobium* faecal 16S real-time PCR Ct values can be seen in Fig 3.

For all three positive *S*. *haematobium* urine-egg output categories (1–10, 11–50 and >50), there was a large *S*. *haematobium* faecal 16S real-time PCR Ct value range and interquartile range. For the negative *S*. *haematobium* urine-egg output category (0), the *S*. *haematobium* faecal 16S real-time PCR Ct value range and interquartile range were relatively much narrower. There were, however, far fewer samples negative by urine-egg microscopy that were positive by *S*. *haematobium* faecal 16S real-time PCR when compared to the number of samples positive by urine-egg microscopy also positive by *S*. *haematobium* faecal 16S real-time PCR (eight and 71, respectively).

The fitted linear model was statistically significant (R^2^ = 0.82, F = 4.2, p = 0.008). All positive *S*. *haematobium* urine-egg output categories were found to significantly predict *S*. *haematobium* faecal 16S real-time PCR Ct values when compared to negative urine-egg microscopy outcomes (Table 5). The one-way ANOVA revealed that there were also statistically significant differences in urine-egg output categories and *S*. *haematobium* faecal real-time PCR Ct values between at least two groups (F = 4.2, p = 0.008). The post hoc Tukey’s HSD test for multiple comparisons found that mean *S*. *haematobium* faecal 16S real-time PCR Ct values were significantly different between negative urine-egg microscopy outcomes and all positive urine-egg microscopy outcome categories (categories 0 and 1–1, categories 0 and 11–50 and categories 0 and > 50). No mean *S*. *haematobium* faecal 16S real-time PCR Ct values were significantly different between all positive urine-egg microscopy outcome categories (categories 1–10 and 11–50, categories 1–10 and >50, and categories 11–50 and > 50, (Table 6)).

**Table 5:** Linear regression correlation coefficient estimates comparing *S*. *haematobium* urine-egg output categories and *S*. *haematobium* faecal 16S real-time PCR Ct values.

*S*. *haematobium* urine-egg output category	Mean difference from intercept (Ct 38.07), (± 95% CI*)	Standard Error	t value	p value
0 (Intercept)	38.07 (34.48–43.66)	/	/	/
1–10	-7.497 (-4.02 - -15.59)	3.111	-2.41	0.026^*‡*^
11–50	-9.277 (-1.29 - -15.59)	3.056	-3.036	0.003^*‡*^
> 50	-9.807 (-3.1 - -15.57)	2.9	-3.382	0.001^*‡*^

Where:

*95% Confidence Intervals

^*‡*^: Statistically significant

^†^: Not statistically significant

**Table 6:** Tukey’s HSD multiple pairwise comparison output comparing *S*. *haematobium* urine-egg output categories and *S*. *haematobium* faecal 16S real-time PCR Ct values.

*S*. *haematobium* urine-egg output categories		Mean difference (± 95% CI*): *S*. *haematobium* faecal 16S real-time PCR Ct value	p value
0	1–10	-7.497 (-1.087 - -15.681)	0.026^*‡*^
0	11–50	-9.277 (-1.237 –-17.316)	0.017^*‡*^
0	> 50	-9.807 (-2.177 –-17.437)	0.006^*‡*^
1–10	11–50	-1.779 (-2.991–6.551)	0.76^†^
1–10	>50	-2.311(-1.733–6.355)	0.44^†^
11–50	>50	-0.53 (-3.211–4.272)	0.98^†^

Where:

*95% Confidence Intervals

^*‡*^: Statistically significant

^†^: Not statistically significant

### 3.3. Molecular characterisation of *Schistosoma* miracidia

#### 3.3.1. *Schistosoma* spp. miracidia mitochondrial *cox*1 and nuclear ITS genotyping

*Cox*1 and ITS sequence data were successfully obtained from 24 miracidia collected from faecal material provided by study participants in both Mchoka and Samama schools. All 24 miracidia collected from Mchoka school were identified as *S*. *mansoni* across both mitochondrial and nuclear regions. All 24 *S*. *mansoni cox*1 and ITS sequences were uploaded to the GenBank repository (Accession numbers: PP545311 –PP545334 and PP544796 –PP544819, respectfully). All 24 miracidia collected from Samama school were identified as *S*. *haematobium* across both mitochondrial and nuclear regions. All 24 *S*. *haematobium cox*1 and ITS sequences were also uploaded to the GenBank repository (Accession numbers: PP545506 –PP545529 and PP545439 –PP545462, respectfully). When examining *S*. *mansoni* and *S*. *haematobium* miracidia ITS chromatograms for double-peaking at species-specific SNP sites, no evidence of mixed-species ancestry was found. In addition, no genetic variation was found between any *S*. *mansoni* miracidia ITS sequence data and no genetic variation was found between any *S*. *haematobium* miracidia ITS sequence data.

#### 3.3.2. Identification of *S*. *mansoni cox*1 lineage groups

**Fig 4:**
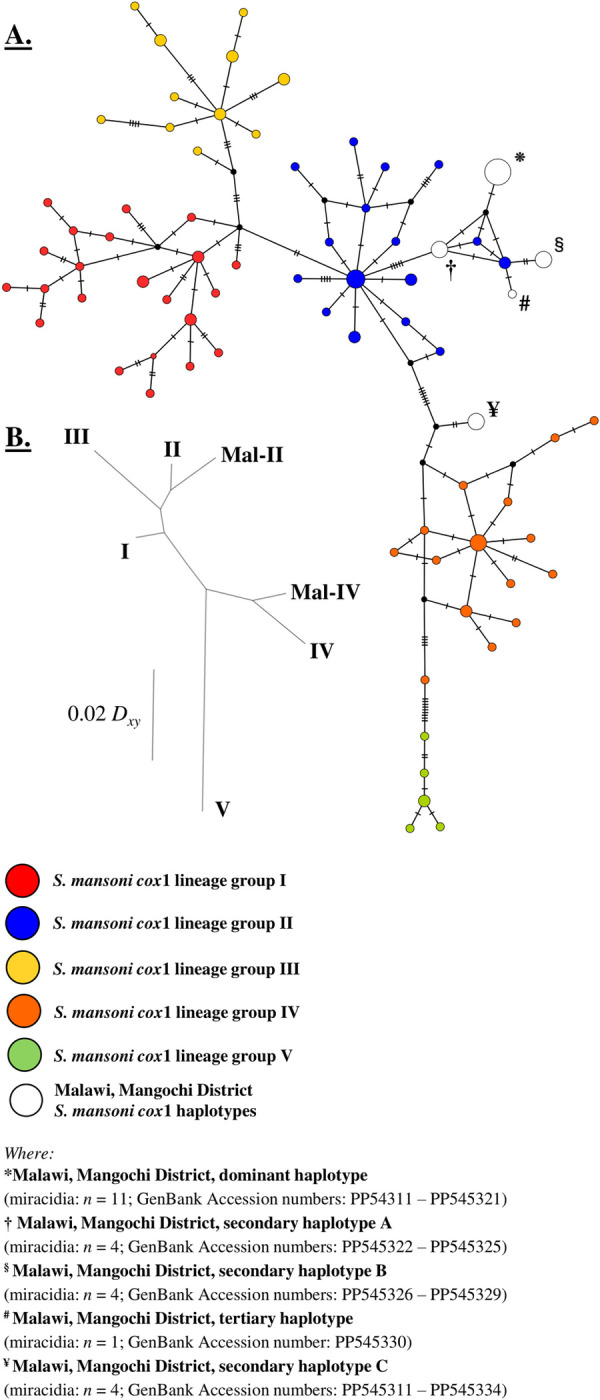
**(A):** TCS haplotype network of *S*. *mansoni cox*1 lineage groups I–V and five Malawi, Mangochi District *S*. *mansoni cox*1 haplotypes. Each node (circle) represents a unique haplotype. Size of nodes is proportional to the frequency of each haplotype. Nodes coloured red denote group I haplotypes (Far west Africa (Senegal and Mali), Brazil, Egypt, Saudi Arabia and Oman), nodes coloured blue denote group II haplotypes (East Africa (Kenya, Uganda, Tanzania and Zambia)), nodes coloured yellow denote group III haplotypes (central west Africa (Cameroon) and Niger), nodes coloured orange denote group IV haplotypes (Zambia and Coastal Kenya) and nodes coloured green denote group V haplotypes (Zambia). Nodes coloured white denote Malawi, Mangochi District haplotypes (*n* = 5) and nodes coloured black denote a missing but predicted ancestral haplotype. Hatched lines denote the number of single nucleotide polymorphisms (SNPs) between nodes. **(B):** Unrooted neighbour joining (NJ) phylogenetic tree constructed to visualise net evolutionary divergence scores between all five *S*. *mansoni cox*1 lineage groups, as well as between Mal-II, and Mal-IV haplotypes. Branch scale length (left) indicates evolutionary divergence distance.

The *S*. *mansoni cox*1 lineage group TCS network can be seen in in Fig 4A. *Cox*1 sequence data from 20 (83.3%) Mangochi District, Malawi *S*. *mansoni* miracidia formed four unique haplotypes that clustered closely to *S*. *mansoni cox*1 lineage group II (geographical topology: East Africa (Kenya, Uganda, Tanzania, and Zambia)). These four haplotypes were therefore grouped within DNAsp and are herein collectively referred to as Mal-II. The degree of evolutionary divergence between Mal-II haplotypes and *S*. *mansoni cox*1 lineage group II (*D*_*xy*_ 0.02183) was less than that between all remaining *S*. *mansoni cox*1 lineage groups (I and III–V; Table 7). The remaining *cox*1 sequence data (from four (16.7%) Mangochi District, Malawi *S*. *mansoni* miracidia) formed one unique haplotype which clustered closely to *S*. *mansoni cox*1 lineage group IV (geographical topology: Zambia and Coastal Kenya). This haplotype was therefore also grouped within DNAsp and is herein referred to as Mal-IV. Again, the degree of evolutionary divergence between this haplotype and *S*. *mansoni cox*1 lineage group IV (*D*_*xy*_ 0.01675) was less than that between all remaining *S*. *mansoni cox*1 lineage groups (I—III and V; Table 7). The unrooted neighbour joining (NJ) phylogenetic tree (Fig 4B) also suggested that Mal-II haplotypes were most closely related to *S*. *mansoni cox*1 lineage group II and Mal-IV haplotypes were most closely related to *S*. *mansoni cox*1 lineage group IV. All Mangochi District, Malawi *S*. *mansoni cox*1 data was uploaded to the GenBank repository (Accession numbers: PP545311 –PP545334).

**Table 7:** Matrices of net evolutionary divergence (*D*_*xy*_) ± standard deviation between previously defined *S*. *mansoni cox*1 lineage groups I–V, Mal-II and Mal-IV. *D*_*xy*_ scores between *S*. *mansoni cox*1 lineage group II and Mal-II, as well as *D*_*xy*_ scores between *S*. *mansoni cox*1 lineage group IV and Mal-IV are highlighted in bold text and underlined.

*S*. *mansoni cox*1 lineage group [16].	I	II	III	IV	V	Mal-II	Mal- IV
**I**	-	-	-	-	-	-	-
**II**	0.02121 ± 0.001	-	-	-	-	-	-
**III**	0.03541 ± 0.002	0.03287 ± 0.002	-	-	-	-	-
**IV**	0.04764 ± 0.004	0.04214 ± 0.003	0.04992 ± 0.003	-	-	-	-
**V**	0.06541 ± 0.006	0.05727 ± 0.004	0.06181 ± 0.005	0.04717 ± 0.004	-	-	-
**Mal-II**	0.03341 ± 0.002	**0.02183** ± 0.002	0.04581 ± 0.003	0.05325 ± 0.004	0.07034 ± 0.007	-	-
**Mal- IV**	0.03958 ± 0.003	0.03458 0.003	0.04995 ± 0.004	**0.01675** ± 0.001	0.0516 ± 0.005	0.0413 ± 0.003	-

## 4. Discussion

In areas of low disease endemicity, highly sensitive diagnostic approaches to identify, diagnose, and monitor schistosomiasis transmission are needed to reliably measure the burden and risk of infection. Here, we used highly sensitive molecular diagnostic methods to gain a thorough understanding of *S*. *mansoni* prevalence and transmission along the southern shoreline of Lake Malawi, Mangochi District, Malawi, five years post-outbreak. This was done to better inform and strengthen future disease control efforts in this area.

Using real-time PCR, intestinal schistosomiasis was confirmed prevalent in school-aged children across the entirety of this shoreline, and, of note, the prevalence of infection was particularly high along its western edge at both Samama and Mchoka schools (55% and 37% prevalence, respectfully). As found elsewhere in other *S*. *mansoni*-endemic areas, the prevalence of infection was much greater using faecal real-time PCR when compared to Kato-Katz faecal microscopy, likely owing to the low-sensitivity of microscopy when attempting to diagnose individuals harbouring low burdens of infection [44,45]. The prevalence of intestinal schistosomiasis by *S*. *mansoni* 16S real-time PCR in each school was reflected by PCR Ct values, suggesting that individuals attending schools with a greater prevalence of infection are also burdened by more heavy infections. Interestingly, however, whilst the prevalence of infection was high across all four schools when using real-time PCR (36% mean prevalence), the burden of infection within individuals appeared to remain generally very low, as measured through the quantification of expelled *S*. *mansoni* eggs using Kato-Katz faecal microscopy. This may be a result of ongoing annual MDA of praziquantel in this area to reduce the transmission of urogenital schistosomiasis, which may be adequate to maintain a low burden of *S*. *mansoni* infections but does not appear adequate to prevent or cease *S*. *mansoni* transmission entirely. Unfortunately, to our knowledge, no previous studies have made any comparisons between *S*. *mansoni* faecal egg count data and *S*. *mansoni* faecal real-time PCR Ct values using this 16S target. Furthermore, as only very few egg-positive Kato-Katz faecal preparations were identified here, and because those that were identified had a mean egg count of just 26.4 epg of faeces, with little variation, no such comparisons can be made using these data. It should also be noted that, based on these analyses, it would appear that the species-specific 16S real-time PCR used here may be less sensitive than the routinely used genus-specific ITS2 real-time PCR as the 16 samples positive by ITS2 real-time PCR, but negative by both *S*. *mansoni* and *S*. *haematobium* 16S real-time PCR, had a mean ITS2 real-time PCR Ct value of 35.7, whereas the mean ITS2 real-time PCR Ct value of all ITS2 positive samples was 28. This, however, requires more investigation to clarify.

As also found previously, the diagnostic performance of POC-CCA compared to molecular diagnosis by PCR varied considerably depending on whether ‘trace’ RDT results were considered a positive or negative diagnostic outcome [28,44,45]. When POC-CCA ‘trace’ results were considered positive, POC-CCA had a sensitivity of 73% and a specificity of 81% when compared to real-time PCR, however, when ‘trace’ results were considered negative, POC-CCA sensitivity was reduced to 56%, whereas specificity was increased to 90%. Whether or not POC-CCA ‘trace’ outcomes should be considered a positive or negative diagnostic outcome should perhaps therefore be contingent on the assay’s intended application, as suggested elsewhere [14]. For example, if being used to measure and map the prevalence of infections within a given community, ‘trace’ results should be considered positive to minimise missed infections leading to an underestimate of disease prevalence, whereas if aiming to confirm *S*. *mansoni* infection on an individual basis, ‘trace’ results should be considered negative to minimise incorrectly diagnosing *S*. *mansoni* infection until a more specific assay has been carried out.

Whilst previous studies have found a strong association between *S*. *mansoni* infection intensity (as measured using Kato-Katz faecal microscopy) and POC-CCA band strength [46], no such association was found here when *S*. *mansoni* infection intensity was estimated using *S*. *mansoni* 16S real-time PCR Ct values. Furthermore, infections missed by POC-CCA that were detected using the *S*. *mansoni* 16S real-time PCR did not appear to be related to the intensity of *S*. *mansoni* infections (again, as inferred using *S*. *mansoni* 16S real-time PCR Ct values). As an example, here, a total of four (1.3%) POC-CCA assays were positive with a band strength indicating heavy infection, however no *S*. *mansoni* eggs were detected in faecal samples provided by these participants, and so all four participants were deemed negative for *S*. *mansoni* infection using microscopy. Whilst the mean *S*. *mansoni* 16S real-time PCR Ct value of these four samples was relatively low compared to the mean Ct of all positive samples (Ct 26.8 and Ct 31, respectively), this was comparatively high when compared to the mean *S*. *mansoni* 16S real-time PCR Ct value of the 10 samples deemed positive by Kato-Katz faecal-egg microscopy (Ct 23.3), which had a mean POC-CCA band strength of 2.4 (moderate infection intensity) but a mean faecal egg count of just 26.4 epg.

There are likely multiple reasons why no association was found between the intensity of *S*. *mansoni* infection (as inferred using *S*. *mansoni* 16S real-time PCR Ct values) and POC-CCA band strength. For example, it has been suggested previously that POC-CCA band strength provides a reliable estimation of *S*. *mansoni* infection intensity only when the average number of *S*. *mansoni* eggs excreted per gram of faeces far exceeds 24, which is not the case here [47]. In addition, it may also be that POC-CCA cross-reactivity with *S*. *haematobium* infection is causing positive (and potentially strong) POC-CCA bands when assessing individuals not infected with *S*. *mansoni* or harbouring *S*. *haematobium* and low-intensity *S*. *mansoni* co-infections. This was suggested by 14 positive POC-CCA results that were negative for *S*. *mansoni* infection by real-time PCR and Kato-Katz faecal-egg microscopy but positive for *S*. *haematobium* infection by urine-egg microscopy (five of which were heavy-intensity *S*. *haematobium* infections, three of which were moderate intensity *S*. *haematobium* infections and six of which were light intensity *S*. *haematobium* infections). This, however, requires further investigation to clarify as the extent to which infection with *S*. *haematobium* can cause a positive POC-CCA outcome, regardless of *S*. *mansoni* infection status, is largely unknown with many studies reporting contradictory data [25,48].

As also found in other *S*. *mansoni* and *S*. *haematobium* co-endemic areas, ectopic *S*. *mansoni* eggs were identified during urine-egg microscopy [49]. Inversely, whilst no ectopic *S*. *haematobium* eggs were identified during Kato-Katz faecal microscopy (which has been found elsewhere [49,50]), a high degree of *S*. *haematobium*-specific mitochondrial 16S DNA was detected in the faecal material of participants infected with *S*. *haematobium*. In addition, motile *S*. *haematobium* miracidia were hatched and recovered from faecal samples.

Multiple explanations for the presence of ectopic *S*. *haematobium* eggs in stool have been proposed. Firstly, this may be caused simply by ‘spill-over’ of *S*. *haematobium* eggs into the lumen of the intestine in individuals harbouring high-intensity *S*. *haematobium* infections [49]. Whilst any spill-over of *S*. *haematobium* eggs in this way cannot be confirmed here, there was a significant negative correlation between *S*. *haematobium* urine-egg output and *S*. *haematobium* faecal 16S real-time PCR Ct values, suggesting that *S*. *haematobium* DNA (either egg-derived or potentially cell-free DNA) is more likely to be detected in faeces provided by individuals harbouring higher-intensity *S*. *haematobium* infections.

It has also been proposed that *S*. *haematobium* eggs may reach the lumen of the intestine when they are deposited by motile *S*. *haematobium* adults in copula during migration from the hepatic portal vein of the liver to the venous plexuses of the bladder [50]. During migration, to reach the venous plexuses of the bladder, *S*. *haematobium* pairings must migrate down the superior rectal (haemorrhoidal) vein and through the middle rectal veins before proceeding to the vesical veins of the bladder. This explanation was proposed following the discovery of unviable *S*. *haematobium* eggs in the rectum and lower sigmoid of the large intestine in 85% of study participants in a *S*. *mansoni* and *S*. *haematobium* co-endemic area [50]. Interestingly, irrespective of whether *S*. *haematobium* eggs expelled in the faeces are viable, egg-derived DNA in the faeces would be detected using real-time PCR, which may explain the detection of *S*. *haematobium* DNA within these faecal samples.

Furthermore, it has also been suggested that the presence of *S*. *haematobium* eggs in stool may be caused by unpartnered *S*. *mansoni* males pairing with *S*. *haematobium* females in the hepatic portal vein and migrating in copula to the to the mesenteric veins of the intestine [49]. Again, whilst eggs produced by such *S*. *mansoni* x *S*. *haematobium* pairings may or may not be viable, the egg-derived maternally inherited mitochondrial DNA would be detected using the *S*. *haematobium* 16S real-time PCR use here, which may also explain the detection of *S*. *haematobium* DNA within these faecal samples. However, it should be noted that no motile *S*. *haematobium* miracidia hatched and recovered from faecal samples here showed any evidence of mixed-species ancestry across the nuclear ITS locus, and so further work is needed to clarify this.

It should also be noted that the detection of *S*. *haematobium* DNA within faecal samples, as well as the recovery of motile *S*. *haematobium* miracidia from faecal samples, may also be caused simply by urine contamination during faecal and urine sample provision. Nevertheless, regardless of the reasons behind the detection of *S*. *haematobium* DNA in faeces, these findings highlight the need for standardised diagnostic assays capable of distinguishing between *Schistosoma* species in multispecies co-endemic areas. Without which, if opting to employ the routinely used genus-specific ITS2 diagnostic real-time PCR using DNA isolated from faecal material, or opting to use the POC-CCA, the prevalence of *S*. *mansoni* infections, as well as the intensity of these infections, may be significantly overestimated.

The identification of five distinct *S*. *mansoni cox*1 haplotypes closely related to *S*. *mansoni cox*1 lineage group II (east Africa (Kenya, Uganda, Tanzania and Zambia)) and group IV (Zambia and Coastal Kenya) suggests multiple recent introduction and colonisation events of *S*. *mansoni* trematodes originating from surrounding east African countries. This recent colonisation of *S*. *mansoni* lineage groups II and IV in Mangochi District likely occurred through human migration, but possibly also through *Biomphalaria* invasion, or both. These findings reflect work carried out previously and further highlight the high degree of mitochondrial *cox*1 genetic diversity both within and between *S*. *mansoni* populations. As recommended previously, the continued monitoring of *Schistosoma* species genetic diversity in endemic populations is needed as this may play an important role in parasite epidemiology, transmission, host-parasite dynamics, host morbidity, and drug efficacy [16].

### Study limitations and future work

Using these data, we were unable to make any robust comparisons between *S*. *mansoni* egg-output and *S*. *mansoni* 16S real-time PCR Ct values as only 2% of all Kato-Katz readings contained ≥ 24 epg. As such, further work should be carried out to assess how *S*. *mansoni* egg-output correlates with *S*. *mansoni* 16S real-time PCR Ct values, allowing for a more accurate estimation of infection intensity when inferred using *S*. *mansoni* 16S Ct values alone.

Further work should also be carried out to develop and validate standardised, highly sensitive, and species-specific diagnostic assays that can also be carried out at the point-of-care in resource-poor schistosomiasis-endemic settings [51,52]. For example, portable and isothermal DNA amplification tools, such as recombinase polymerase amplification (RPA) and loop-mediated isothermal amplification (LAMP), may offer the ability to reliably detect and distinguish between low intensity *S*. *mansoni* and/or *S*. *haematobium* infections in low prevalence areas [53,54]. Currently, however, these assays require further development and validation before their routine and standardised use in disease-endemic areas.

## 5. Conclusions

Using highly sensitive molecular diagnostic approaches, we confirm that intestinal schistosomiasis is highly prevalent across the southern shoreline of Lake Malawi, Mangochi District, Malawi, just five years post initial disease outbreak and despite ongoing annual MDA using praziquantel. In addition, a high prevalence of urogenital schistosomiasis persists, again despite ongoing MDA. As such, the revision of ongoing schistosomiasis control programmes in this area, such as the implementation of bi-annual MDA using praziquantel, significantly improving access to adequate WASH infrastructure, freshwater snail population control, and further delivery of schistosomiasis education and health programmes to promote behaviours that limit the risk of contracting and transmitting schistosomiasis is therefore recommended in line with WHO recommendations. Our study also highlights the urgent need for highly sensitive and standardised diagnostic assays capable of distinguishing between *Schistosoma* species in low prevalence multispecies co-endemic areas. The further development of diagnostic tools able to detect and distinguish between low-intensity multispecies *Schistosoma* infections, that can also be carried out in resource-poor schistosomiasis-endemic settings, is therefore also strongly encouraged.

## Supporting information

S1 File Molecular methods. (DOCX)

S2 File *Schistosoma mansoni* reference sequences and GenBank accession numbers used for phylogenetic analyses. (DOCX)

S1 Data Diagnostic and genotyping data. (XLSX)
