# Supplementary material for: Molecular epidemiology and population genetics of Schistosoma mansoni infecting school-aged children situated along the southern shoreline of Lake Malawi, Malawi
Source: PLoS Negl Trop Dis. 2024 Oct 7;18(10):e0012504. doi: 10.1371/journal.pntd.0012504 (PMC11458004; doi:10.1371/journal.pntd.0012504)
Supplement: S1 File — (DOCX) [file pntd.0012504.s001.docx]

Molecular epidemiology and population genetics of *Schistosoma mansoni* infecting school-aged children situated along the southern shoreline of Lake Malawi, Mangochi District, Malawi

**Supplemental file 1: Molecular methods**

*Genus-specific* Schistosoma *spp. ITS2 rDNA real-time PCR*

**Table A:** Primer and probe sequences used to amplify a 77 bp fragment of the genus-specific *Schistosoma* spp. internal transcribed spacer 2 (ITS2) ribosomal DNA (rDNA) region and an 89 bp fragment of the PhHV-1 glycoprotein B gene [1].

| **Name** | **Target** | **Oligonucleotide sequence (5’ – 3’)** | **Reference** |
| --- | --- | --- | --- |
| **Sch_ITS_F*** | *Schistosoma* spp. ITS2 | GGTCTAGATGACTTGATYGAGATGCT | [1]. |
| **Sch_ITS_R**^†^ |  | TCCCGAGCGYGTATAATGTCATTA |  |
| **Sch_ITS_Pro**^‡^ |  | (**FAM^¥^**) TGGGTTGTGCTCGAGTCGTGGC |  |
| **PhHV-1-267s*** | PhHV-1 glycoprotein B | GGGCGAATCACAGATTGAATC |  |
| **PhHV-1-337as**^†^ |  | GCGGTTCCAAACGTACCAA |  |
| **PhHV-1-305tq**^‡^ |  | (**Cy5^¥^**) TTTTTATGTGTCCGCCACCATCTGGATC |  |

**Forward primer*

^†^*Reverse primer*

*^‡^Probe*

^¥^*Fluorophore*

**Table B:** Reaction mix used to carry out a duplex real-time PCR targeting a 77 bp fragment of the genus-specific *Schistosoma* spp. internal transcribed spacer 2 (ITS2) ribosomal DNA (rDNA) region and an 89 bp fragment of the PhHV-1 glycoprotein B gene [1].

|  | **Concentration** | **Volume per 1 sample (µl)** |
| --- | --- | --- |
| ddH_2_O | / | 6.2 |
|  |  |  |
| Sch_ITS_F* | 2.5 pmol | 0.25 |
| Sch_ITS_R ^†^ | 2.5 pmol | 0.25 |
| Sch_ITS_Pro ^‡^ | 2.5 pmol | 0.25 |
|  |  |  |
| PhHV-1-267s * | 1.5 pmol | 0.15 |
| PhHV-1-337as ^†^ | 1.5 pmol | 0.15 |
| PhHV-1-305tq^‡^ | 2.5 pmol | 0.25 |
|  |  |  |
| AppPROBE No ROX mix (Appleton Woods, UK) | / | 12.5 |
|  |  |  |
| DNA | / | 5 |
|  |  | **25** |

**Forward primer*

^†^*Reverse primer*

*^‡^Probe*

**Table C:** Cycling conditions used to carry out a duplex real-time PCR targeting a 77 bp fragment of the genus-specific *Schistosoma* spp. internal transcribed spacer 2 (ITS2) region and an 89 bp fragment of the PhHV-1 glycoprotein B gene [1].

| **PCR step** | **Time** | **Temp** | **Cycles** |
| --- | --- | --- | --- |
| Denaturation | 3 min | 95 ºC | / |
| Annealing | 10 sec | 95 ºC | 40 |
|  | 30 sec | 60 ºC |  |

*Species-specific* S. mansoni *and* S. haematobium *mitochondrial 16s rDNA real-time PCR*

**Table D:** Primer and probe sequences used to amplify a 104 bp species-specific fragment of the *S. mansoni* mitochondrial 16S ribosomal DNA (rDNA) and a 143 bp species-specific fragment of the *S. haematobium* 16S rDNA [2].

| **Name** | **Target** | **Oligonucleotide sequence (5’ – 3’)** | **Reference** |
| --- | --- | --- | --- |
| **Sm_16S_FW*** | *S. mansoni* 16S locus | CTGCTCAGTGAAGAAGTTTGTTT | [2]. |
| **Sm_16S_RV**^†^ |  | CCTCATTGAACCATTCACAAGTC |  |
| **Sm_16S_Pro**^‡^ |  | (**FAM^¥^**) AGCCGCGATTATTTATCGTGCTAAGGT |  |
| **Sh_16S_FW*** | *S. haematobium* 16S locus | AATGAACATGAATGGCCGCA |  |
| **Sh_16S_RV**^†^ |  | ATGGGTTCCTCACCACTTAAACT |  |
| **Sh_16S_Pro**^‡^ |  | (**HEX^¥^**) TGGAGACTTGTGAATGGTCGAACG |  |

**Forward primer*

^†^*Reverse primer*

*^‡^Probe*

^¥^*Fluorophore*

**Table E:** Reaction mix used to carry out a duplex real-time PCR targeting a 104 bp species-specific fragment of the *S. mansoni* mitochondrial 16S ribosomal DNA (rDNA) and a 143 bp species-specific fragment of the *S. haematobium* 16S rDNA [2].

|  | **Concentration** | **Volume per 1 sample (µl)** |
| --- | --- | --- |
| ddH_2_O | / | 5.7 |
|  |  |  |
| Sm_16S_FW* | 2.5 pmol | 0.25 |
| Sm_16S_RV^†^ | 2.5 pmol | 0.25 |
| Sm_16S_Pro^‡^ | 2.5 pmol | 0.25 |
|  |  |  |
| Sh_16S_FW* | 4 pmol | 0.4 |
| Sh_16S_RV^†^ | 4 pmol | 0.4 |
| Sh_16S_Pro^‡^ | 2.5 pmol | 0.25 |
|  |  |  |
| AppPROBE No ROX mix (Appleton Woods, UK) | / | 12.5 |
|  |  |  |
| DNA | / | 5 |
|  |  | **25** |

**Forward primer*

^†^*Reverse primer*

*^‡^Probe*

**Table F:** Cycling conditions used to carry out a duplex real-time PCR targeting a 104 bp species-specific fragment of the *S. mansoni* mitochondrial 16S ribosomal DNA (rDNA) and a 143 bp species-specific fragment of the *S. haematobium* 16S rDNA [2].

| **PCR step** | **Time** | **Temp** | **Cycles** |
| --- | --- | --- | --- |
| Denaturation | 3 min | 95 ºC | / |
| Annealing | 10 sec | 95 ºC | 45 |
|  | 30 sec | 60 ºC |  |

*Species-specific* S. mansoni *and* S. haematobium *mitochondrial* cox*1 rDNA end-point PCR and Sanger sequencing*

**Table G:** Primer sequences used to amplify a partial fragment of the *Schistosoma* spp. mitochondrial cytochrome oxidase subunit 1 (*cox*1) gene. This species-specific PCR uses one universal forward primer and four distinct reverse primers specific to *S. mansoni, S. haematobium, S. mattheei* and *S. bovis* to amplify four *cox*1 regions that differ in length according to *Schistosoma* species (375 bp, 543 bp, 362 bp and 306 bp, respectively), [3].

| **Name** | **Species** | **Oligonucleotide sequence (5’ – 3’)** | **Reference** |
| --- | --- | --- | --- |
| **Asmit1_FW*** | Universal | TTTTTTGGTCATCCTGAGGTGTAT | [3]. |
| **Sman_RV**^†^ | *S. mansoni* | TGCAGATAAAGCCACCCCTGTG |  |
| **Sh_RV**^†^ | *S. haematobium* | TGATAATCAATGACCCTGCAATAA |  |
| **Smat_RV**^†^ | *S. matthei* | CACCAGTTACACCACCAACAGA |  |
| **Sb_RV**^†^ | *S. bovis* | CACAGGATCAGACAAACGAGTACC |  |

**Forward primer*

^†^*Reverse primer*

**Table H:** Reaction mix used to carry out a multiplex endpoint PCR targeting a partial fragment of the *Schistosoma* spp. mitochondrial cytochrome oxidase subunit 1 (*cox*1) gene. This species-specific PCR uses one universal forward primer and four distinct reverse primers specific to *S. mansoni, S. haematobium, S. mattheei* and *S. bovis* to amplify four *cox*1 regions that differ in length according to *Schistosoma* species (375 bp, 543 bp, 362 bp and 306 bp, respectively), [3].

|  | **Concentration** | **Volume per 1 sample (µl)** |
| --- | --- | --- |
| ddH_2_O | / | 18 |
|  |  |  |
| Asmit1_FW* | 20 pmol | 2 |
| Sman_RV^†^ | 7.5 pmol | 0.75 |
| Sh_RV^†^ | 7.5 pmol | 0.75 |
| Smat_RV^†^ | 7.5 pmol | 0.75 |
| Sb_RV^†^ | 7.5 pmol | 0.75 |
|  |  |  |
| Illustra PuReTaq ready-to-go PCR bead  [Sigma-Aldrich, USA] | / | **/** |
|  |  |  |
| DNA | / | **2** |
|  |  | **25** |

**Forward primer*

^†^*Reverse primer*

**Table I:** PCR conditions used to carry out a multiplex endpoint PCR targeting a partial fragment of the *Schistosoma* spp. mitochondrial cytochrome oxidase subunit 1 (*cox*1) gene. This species-specific PCR uses one universal forward primer and four distinct reverse primers specific to *S. mansoni, S. haematobium, S. mattheei* and *S. bovis* to amplify four *cox*1 regions that differ in length according to *Schistosoma* species (375 bp, 543 bp, 362 bp and 306 bp, respectively), [3].

| **PCR step** | **Time** | **Temp** | **Cycles** |
| --- | --- | --- | --- |
| Denaturation | 3 minutes | 95 | / |
| Annealing | 30 seconds | 94 | 40 |
|  | 45 seconds | 62 |  |
|  | 45 seconds | 72 |  |
| Extension | 10 minutes | 72 | / |

*Mitochondrial* cox*1 genotyping:* Schistosoma *spp. miracidia*

**Table J:** Primer and probe sequences used to amplify a 956 bp region of the *Schistosoma* spp. mitochondrial cytochrome oxidase subunit 1 (*cox*1) gene [4].

| **Name** | **Target** | **Oligonucleotide sequence (5’ – 3’)** | **Reference** |
| --- | --- | --- | --- |
| **Schisto_5’*** | *Schistosoma* spp. *cox*1 locus | TCTTTRGATCATAAGCG | [4]. |
| **Schisto_3’**^†^ |  | TAATGCATMGGAAAAAAACA |  |

**Forward primer*

^†^*Reverse primer*

**Table K:** Reaction mix used to carry out end-point targeting a 956 bp region of the *Schistosoma* spp. mitochondrial cytochrome oxidase subunit 1 (*cox*1) gene [4].

|  | **Concentration** | **Volume per 1 sample (µl)** |
| --- | --- | --- |
| ddH_2_O | / | 20 |
|  |  |  |
| Schisto_5’* | 10 pmol | 1 |
| Schisto_3’^†^ | 10 pmol | 1 |
|  |  |  |
| Illustra PuReTaq ready-to-go PCR bead  [Sigma-Aldrich, USA] | / | **/** |
|  |  |  |
| DNA | / | 3 |
|  |  | **25** |

**Forward primer*

^†^*Reverse primer*

**Table L:** PCR conditions used to carry out end-point targeting a 956 bp region of the *Schistosoma* spp. mitochondrial cytochrome oxidase subunit 1 (*cox*1) gene [4].

| **PCR step** | **Time** | **Temp** | **Cycles** |
| --- | --- | --- | --- |
| Denaturation | 5 min | 95 ºC | / |
| Annealing | 30 sec | 95 ºC | 40 |
|  | 30 sec | 40 ºC |  |
|  | 1.30 min | 72 ºC |  |
| Extension | 10 min | 72 ºC | / |

*Nuclear ITS genotyping:* Schistosoma *spp. miracidia*

**Table M:** Primer and probe sequences used to amplify the complete *Schistosoma* spp. nuclear internal transcribed spacer region (inclusive of both ITS regions 1 + 2 and the nuclear 5.8S region) [5].

| **Name** | **Target** | **Oligonucleotide sequence (5’ – 3’)** | **Reference** |
| --- | --- | --- | --- |
| **ETTS2*** | *Schistosoma* spp. ITS locus | TAACAAGGTTTCCGTAGGTGA | [5]. |
| **ETTS1**^†^ |  | TGCTTAAGTTCAGCGGG |  |

**Forward primer*

^†^*Reverse primer*

**Table N:** Reaction mix used to carry out end-point PCR targeting the complete *Schistosoma* spp. nuclear internal transcribed spacer region (inclusive of both ITS regions 1 + 2 and the nuclear 5.8S region) [5].

|  | **Concentration** | **Volume per 1 sample (µl)** |
| --- | --- | --- |
| ddH_2_O | / | 20 |
|  |  |  |
| ETTS2* | 10 pmol | 1 |
| ETTS1^†^ | 10 pmol | 1 |
|  |  |  |
| Illustra PuReTaq ready-to-go PCR bead  [Sigma-Aldrich, USA] | / | **/** |
|  |  |  |
| DNA | / | **3** |
|  |  | **25** |

**Forward primer*

^†^*Reverse primer*

**Table O:** PCR conditions used to carry out end-point PCR targeting the complete *Schistosoma* spp. nuclear internal transcribed spacer region (inclusive of both ITS regions 1 + 2 and the nuclear 5.8S region) [5].

| **PCR step** | **Time** | **Temp** | **Cycles** |
| --- | --- | --- | --- |
| Denaturation | 5 min | 95 ºC | / |
| Annealing | 30 sec | 95 ºC | 40 |
|  | 30 sec | 58 ºC |  |
|  | 1.30 min | 72 ºC |  |
| Extension | 10 min | 72 ºC | / |

**References**

1. Armoo S, Cunningham LJ, Campbell SJ, Aboagye FT, Boampong FK, Hamidu BA, *et al*. Detecting *Schistosoma mansoni* infections among pre-school-aged children in southern Ghana: A diagnostic comparison of urine-CCA, real-time PCR and Kato-Katz assays. *BMC Infectious Diseases*. 2020;20: 1–10. doi:10.1186/s12879-020-05034-2.

2. Alzaylaee H, Collins RA, Rinaldi G, Shechonge A, Ngatunga B, Morgan ER, *et al*. *Schistosoma* species detection by environmental DNA assays in African freshwaters. *PLoS Neglected Tropical Diseases*. 2020;14: 1–19. doi:10.1371/journal.pntd.0008129.

3. Schols R, Carolus H, Hammoud C, Mulero S, Mudavanhu A, Huyse T. A rapid diagnostic multiplex PCR approach for xenomonitoring of human and animal schistosomiasis in a ‘One Health’ context. *Transactions of The Royal Society of Tropical Medicine and Hygiene*. 2019;113: 722–729. doi:10.1093/trstmh/trz067.

4. Lockyer AE, Olson PD, Østergaard P, Rollinson D, Johnston DA, Attwood SW, *et al*. The phylogeny of the Schistosomatidae based on three genes with emphasis on the interrelationships of *Schistosoma* Weinland, 1858. *Parasitology*. 2003;126: 203–224. doi:10.1017/S0031182002002792.

5. Kane RA, Ridgers IL, Johnston DA, Rollinson D. Repetitive sequences within the first internal transcribed spacer of ribosomal DNA in schistosomes contain a Chi-like site. *Molecular and Biochemical Parasitology*. 1996;75: 265–269. doi:10.1016/0166-6851(95)02525-1.
