# Supplementary material for: Molecular epidemiology and population genetics of Schistosoma mansoni infecting school-aged children situated along the southern shoreline of Lake Malawi, Malawi
Source: PLoS Negl Trop Dis. 2024 Oct 7;18(10):e0012504. doi: 10.1371/journal.pntd.0012504 (PMC11458004; doi:10.1371/journal.pntd.0012504)
Supplement: S2 File — (DOCX) [file pntd.0012504.s002.docx]

Molecular epidemiology and population genetics of *Schistosoma mansoni* infecting school-aged children situated along the southern shoreline of Lake Malawi, Mangochi District, Malawi

**Supplemental file 2: *Schistosoma mansoni* reference sequences and GenBank accession numbers used for phylogenetic analyses.**

**Table A:** *S. mansoni cox*1 lineage group I – V [1] reference sequences and GenBank accession numbers used to identify which lineage group Mangochi District *S. mansoni cox*1 sequences were most closely related to.

| ***S. mansoni cox*1 lineage group** [1]**.** | **Country (location)** | **GenBank accession numbers** |
| --- | --- | --- |
| **I** | \| Niger (Diambala) \| \| --- \| \| Niger (Diambala) \| \| Niger (Diambala) \| \| Niger (Diambala) \| \| Niger (Namarigoungou) \| \| Niger (Namarigoungou) \| \| Niger (Namarigoungou) \| \| Niger (Namarigoungou) \| \| Mali (Farako) \| \| Egypt (unknown) \| \| Senegal (Temeye) \| \| Senegal (Temeye) \| \| Senegal (Temeye) \| \| Senegal (Temeye) \| \| Senegal (Temeye) \| \| Senegal (Nder) \| \| Senegal (Nder) \| \| Senegal (Nder) \| \| Senegal (Nder) \| \| Brazil (Ndombo) \| \| Brazil (Belo Horizonte) \| \| Oman (unknown) \| \| Saudi Arabia (Medina) \| | \| JQ289647 \| \| --- \| \| JQ289645 \| \| JQ289648 \| \| JQ289644 \| \| JQ289637 \| \| JQ289624 \| \| JQ289631 \| \| JQ289628 \| \| JQ289621 \| \| JQ289620 \| \| JQ289682 \| \| JQ289687 \| \| JQ289683 \| \| JQ289678 \| \| JQ289688 \| \| JQ289674 \| \| JQ289667 \| \| JQ289671 \| \| JQ289670 \| \| JQ289658 \| \| JQ289587 \| \| JQ289651 \| \| JQ289653 \| |
| **II** | \| Kenya (unknown) \| \| --- \| \| Kenya (unknown) \| \| Kenya (Rekeke District) \| \| Kenya (Rekeke District) \| \| Kenya (Rekeke District) \| \| Kenya (Rekeke District) \| \| Uganda (Walakuba District) \| \| Uganda (Walakuba District) \| \| Uganda (Walakuba District) \| \| Uganda (Butiaba) \| \| Uganda (Butiaba) \| \| Uganda (Butiaba) \| \| Uganda (Butogo) \| \| Uganda (Butogo) \| \| Uganda (Butogo) \| \| Zambia (Siamikobo) \| \| Zambia (Siamikobo) \| \| Zambia (Siamikobo) \| \| Tanzania (Hamuyebe District) \| \| Tanzania (Hamuyebe District) \| \| Tanzania (Hamuyebe District) \| \| Tanzania (Hamuyebe District) \| \| Tanzania (Hamuyebe District) \| | \| JQ289618 \| \| --- \| \| JQ289619 \| \| JQ289613 \| \| JQ289608 \| \| JQ289601 \| \| JQ289599 \| \| JQ289724 \| \| JQ289722 \| \| JQ289721 \| \| JQ289719 \| \| JQ289716 \| \| JQ289720 \| \| JQ289713 \| \| JQ289714 \| \| JQ289712 \| \| JQ289739 \| \| JQ289740 \| \| JQ289741 \| \| JQ289698 \| \| JQ289694 \| \| JQ289703 \| \| JQ289692 \| \| JQ289708 \| |
| **III** | \| Niger (Namarigoungou) \| \| --- \| \| Niger (Namarigoungou) \| \| Niger (Namarigoungou) \| \| Niger (Diambala) \| \| Niger (Diambala) \| \| Nigeria (Nebbi District) \| \| Nigeria (Nebbi District) \| \| Cameroon (Bessoum) \| \| Cameroon (Bessoum) \| \| Cameroon (Bessoum) \| \| Cameroon (Bessoum) \| \| Cameroon (Bessoum) \| \| Cameroon (Bessoum) \| \| Cameroon (Bessoum) \| \| Cameroon (Bessoum) \| | \| JQ289639 \| \| --- \| \| JQ289640 \| \| JQ289630 \| \| JQ289649 \| \| JQ289646 \| \| JQ289622 \| \| JQ289623 \| \| JQ289588 \| \| JQ289590 \| \| JQ289594 \| \| JQ289592 \| \| JQ289595 \| \| JQ289593 \| \| JQ289589 \| \| JQ289591 \| |
| **IV** | \| Zambia (Kaunga District) \| \| --- \| \| Zambia (Kaunga District) \| \| Zambia (Kaunga District) \| \| Zambia (Kaunga District) \| \| Zambia (Kaunga District) \| \| Kenya (Rekeke) \| \| Kenya (Rekeke) \| \| Kenya (Rekeke) \| \| Kenya (Rekeke) \| \| Kenya (Rekeke) \| \| Kenya (Rekeke) \| \| Kenya (Rekeke) \| \| Kenya (Rekeke) \| \| Kenya (Rekeke) \| \| Kenya (Rekeke) \| \| Kenya (Rekeke) \| \| Kenya (Rekeke) \| \| Kenya (Rekeke) \| | \| JQ289734 \| \| --- \| \| JQ289729 \| \| JQ289733 \| \| JQ289738 \| \| JQ289736 \| \| JQ289603 \| \| JQ289615 \| \| JQ289607 \| \| JQ289614 \| \| JQ289605 \| \| JQ289617 \| \| JQ289609 \| \| JQ289597 \| \| JQ289611 \| \| JQ289616 \| \| JQ289598 \| \| JQ289604 \| \| JQ289610 \| |
| **V** | \| Zambia (Kaunga District) \| \| --- \| \| Zambia (Kaunga District) \| \| Zambia (Kaunga District) \| \| Zambia (Kaunga District) \| \| Zambia (Kaunga District) \| \| Zambia (Kaunga District) \| \| Zambia (Kaunga District) \| | \| JQ289731 \| \| --- \| \| JQ289730 \| \| JQ289737 \| \| JQ289735 \| \| JQ289732 \| \| JQ289728 \| \| JQ289727 \| |

**References**

1. Webster BL, Webster JP, Gouvras AN, Garba A, Lamine MS, Diaw OT, *et al*. DNA ‘barcoding’ of *Schistosoma mansoni* across sub-Saharan Africa supports substantial within locality diversity and geographical separation of genotypes. *Acta Tropica*. 2013;128: 250–260. doi:10.1016/j.actatropica.2012.08.009.
